# lncRNA1386.1/novel-miR0032-5p Axis Targets *CAT* Gene to Modulate Redox and Immune Reponses of *Apis cerana* Larvae to *Ascosphaera apis* Infection

**DOI:** 10.3390/antiox15080959

**Published:** 2026-07-31

**Authors:** Xiaoxue Fan, Nian Fan, Kunze Li, Kaiyao Zhang, Xue Yang, Jiarun Yang, Jing Tian, Jianfeng Qiu, Qingwei Tan, Dafu Chen, Rui Guo

**Affiliations:** 1 College of Bee Science, Fujian Agriculture and Forestry University, Fuzhou 350002, China; imfanxx@163.com (X.F.); 13595105054@163.com (N.F.); kunze0515@163.com (K.L.); kaiyao1223@126.com (K.Z.); y2745860171@163.com (X.Y.); 13064033836@163.com (J.Y.); 18064856039@163.com (J.T.); jfqiu@fafu.edu.cn (J.Q.); tanqingwei@fafu.edu.cn (Q.T.); 2 National & Local United Engineering Laboratory of Natural Biotoxin, Fujian Agriculture and Forestry University, Fuzhou 350002, China; 3 Apitherapy Research Institute, Fujian Agriculture and Forestry University, Fuzhou 350002, China

**Keywords:** honey bee, *Apis cerana*, *Ascosphaera apis*, non-coding RNA, lncRNA, competing endogenous RNA

## Abstract

Long non-coding RNAs (lncRNAs) participate in insect immune regulation, but their relationship with antioxidant responses during fungal infection remains unclear. Here, we examined a candidate regulatory relationship among lncRNA1386.1, novel-miR0032-5p, and the catalase gene (*CAT*) in *Apis cerana* worker larvae infected with *Ascosphaera apis*. Dual-luciferase reporter assays showed that M-miR0032-5p reduced the activity of reporters containing the predicted miRNA response element within lncRNA1386.1 or *CAT*, whereas mutation of these sequences weakened or abolished the response. In infected larvae, lncRNA1386.1 silencing and novel-miR0032-5p overexpression reduced *CAT* transcript abundance and CAT protein concentration, and increased dihydroethidium (DHE) fluorescence intensity. These treatments were also accompanied by alterations in the transcript abundance of the host genes *Dorsal*1 and *Relish* and the fungal genes *Chit*3 and *STE*11*-like*. Novel-miR0032-5p inhibition generally produced opposite changes in CAT-related measurements, DHE fluorescence intensity, and the selected host and fungal transcripts. lncRNA1386.1 silencing was associated with increased larval survival, whereas novel-miR0032-5p inhibition was associated with a higher hazard of first visible external mycelial growth without significantly affecting survival. These findings support a negative regulatory role of novel-miR0032-5p in *CAT* expression and indicate that lncRNA1386.1 participates in a candidate shared miRNA-responsive regulatory relationship. This candidate regulatory relationship was associated with CAT-related antioxidant regulation, superoxide-associated DHE fluorescence, immune-related transcription, selected fungal transcriptional responses, and visible external mycelial growth during *A. apis* infection.

## 1. Introduction

*Apis cerana* is native to much of Asia and has long been managed for honey production and pollination services [1]. Chalkbrood, caused by the fungal pathogen *Ascosphaera apis*, is a fatal brood disease associated with larval mortality and impaired colony development [2]. Infection begins when susceptible larvae ingest fungal spores, which germinate in the gut and subsequently invade larval tissues as hyphae [3,4]. Disease development is favored by unfavorable brood-rearing conditions, including high humidity and abrupt changes in temperature [3]. Previous studies have reported miRNA-mediated immune regulation and changes in antioxidant enzyme activities during *A. apis* infection [5,6]. However, how non-coding RNA regulation is linked to antioxidant responses and infection-associated physiological stress in *A. cerana* larvae remains unclear.

Insects rely primarily on innate immunity, which includes cellular and humoral responses [7]. The Toll signaling pathway is an essential component of antifungal defense and is activated through pathogen recognition and Spätzle-dependent signaling, leading to NF-κB-mediated expression of antimicrobial peptides [8,9,10]. In honey bees, which lack a *Dif* ortholog, *Dorsal*1 and *Dorsal*2 participate in the regulation of antimicrobial peptides such as Defensin-1 [11]. The Imd signaling pathway is mainly associated with responses to Gram-negative bacteria and activates the NF-κB transcription factor *Relish*, which regulates antimicrobial peptides including Abaecin and Hymenoptaecin [12]. *Dorsal*1 and *Relish* therefore represent pivotal immune-related transcriptional markers for assessing host responses to infection. These immune-relevant transcriptional responses may also interact with broader physiological processes, including oxidative stress regulation and tissue homeostasis during host–pathogen interactions [13,14].

Reactive oxygen species (ROS) production is a vital component of antimicrobial defense in insects [15]. However, excessive ROS can damage host lipids, proteins, and DNA, necessitating antioxidant defenses to maintain redox homeostasis [16]. Among key antioxidant enzymes, catalase (CAT) plays a pivotal role in detoxifying hydrogen peroxide (H_2_O_2_) [17]. In honey bee larvae, *A. apis* infection has been associated with altered antioxidant defenses. In *A. cerana*, infection reduced SOD and CAT activities in the guts of 5- and 6-day-old larvae and caused stage-dependent changes in GST and PPO activities [6]. In *Apis mellifera*, infected larvae showed lower SOD, CAT, and GST activities, together with metabolomic changes consistent with oxidative stress [18]. ROS may also interact with immune-related signaling, linking oxidative status with host defense. However, the post-transcriptional mechanisms connecting non-coding RNAs, *CAT* regulation, and infection-associated redox responses in *A. cerana* larvae remain unclear.

Non-coding RNAs (ncRNAs) add a further regulatory layer to insect immunity [19]. MicroRNAs (miRNAs) fine-tune immune gene output by directing RISC to complementary target sites, typically within the 3′ untranslated region (3′ UTR), thus repressing translation or promoting mRNA decay [20]. Consistent with this role, multiple miRNAs have been linked to immune modulation across insects, including miR-210 in *D. melanogaster* and miR-184 in hemipterans [21,22]. In *A. cerana* worker larvae challenged by *A. apis*, gut miRNA profiles change dynamically, and ace-miR-539-y is involved in regulating the JAK-STAT signaling pathway and phagocytosis-related genes during early responses [23]. Long non-coding RNAs (lncRNAs) also contribute to immune regulation through *cis*/*trans* actions or by interfacing with post-transcriptional control [24]. Thousands of lncRNAs have been cataloged in honey bee larval guts, many of which respond transcriptionally to *A. apis* infection [25]. Some lncRNAs may act as miRNA precursors or influence miRNA-mediated regulation by sharing miRNA response elements with target transcripts. Competing endogenous RNA (ceRNA) activity depends on the relative abundance and subcellular localization of the interacting RNAs, as well as their association with Argonaute-containing RISC. Accordingly, the lncRNA1386.1/novel-miR0032-5p/*CAT* relationship was examined as a candidate shared miRNA-responsive axis [26,27,28,29].

Transcriptomic analyses of *A. cerana* larval guts have identified infection-associated changes in immune- and metabolism-associated gene expression [30]. Small-RNA sequencing has also revealed infection-responsive miRNAs whose predicted targets are enriched in immune-related processes, including ubiquitin-mediated proteolysis and JAK-STAT signaling [23]. Strand-specific RNA sequencing further identified differentially expressed lncRNAs and supported the construction of predicted lncRNA-miRNA-mRNA regulatory networks [25]. These networks included candidate relationships linking immune-related processes, such as endocytosis, phagocytosis, and MAPK signaling, with antioxidant regulation [31].

In our previous integrative analysis, infection-responsive lncRNAs, miRNAs, and mRNAs were linked using RNAhybrid, miRanda, and TargetScan. The lncRNA1386.1/novel-miR0032-5p/*CAT* relationship was selected from an antioxidant-associated subnetwork since lncRNA1386.1 was differentially expressed following *A. apis* infection and both lncRNA1386.1 and *CAT* contained predicted miRNA response elements (MREs) for novel-miR0032-5p. The present study extended these omics-based predictions by testing the predicted response sequences using dual-luciferase reporters and by separately manipulating lncRNA1386.1 and novel-miR0032-5p in infected larvae.

Therefore, this study examined the candidate regulatory relationship among lncRNA1386.1, novel-miR0032-5p, and *CAT* in the guts of *A. cerana* worker larvae during *A. apis* infection. We combined expression profiling, dual-luciferase reporter assays, lncRNA1386.1 silencing, and novel-miR0032-5p overexpression or inhibition to evaluate whether the three RNAs showed sequence-responsive and expression relationships compatible with a shared miRNA-responsive model. We further measured *CAT* transcript abundance and CAT protein concentration, dihydroethidium (DHE) fluorescence intensity, host *Dorsal*1 and *Relish* transcript abundance, fungal *Chit*3 and *STE*11*-like* transcript abundance, larval survival, and chalkbrood-associated outcomes. This study provides experimental support for linking non-coding RNA regulation with CAT-related redox responses, immune-related transcription, and host-fungus interactions and offers candidate molecular indicators for future studies of chalkbrood susceptibility and resistance.

## 2. Materials and Methods

### 2.1. Honey Bee and Fungal Pathogen

*A. cerana* worker larvae were obtained from three colonies reared in the teaching apiary of the College of Bee Science, Fujian Agriculture and Forestry University. The *A. apis* strain was isolated by our lab and deposited at the China General Microbiological Culture Collection Center under accession number: 40895.

### 2.2. Experimental Design, Randomization, Blinding, and ARRIVE Compliance

For the molecular, fluorescence-imaging, and CAT protein assays, three pooled gut samples were analyzed as biological replicates for each experimental group at each sampling time. Each pooled sample comprised gut tissues from three larvae originating from a single source colony, resulting in three biological replicates and nine larvae per group at each dpi. Larvae within each pooled sample were treated as subsamples rather than independent observations. For the survival and visible external mycelial-growth assays, each larva was maintained individually and treated as the experimental unit. Each group contained 48 larvae originating from the three source colonies, with 16 larvae contributed by each colony. All molecular, biochemical, fluorescence-imaging, survival, and visible external mycelial-growth assays were conducted within a single experimental run.

Only healthy, normally developing worker larvae of the same developmental stage were included. Larvae showing abnormal morphology, mechanical injury during grafting, failure to consume the diet, contamination, or death before treatment allocation were excluded according to criteria established before the experiments. No larvae or data points were excluded after treatment allocation unless otherwise stated.

The sample size was determined based on our previous studies using the same larval infection model and the feasibility of obtaining sufficient biological replicates. No formal a priori sample-size or statistical-power calculation was performed.

Larvae were distributed among treatment groups and plate positions in a balanced manner to minimize potential effects associated with plate position and handling order. Investigators performing larval inoculation and group-specific feeding were aware of treatment allocation because different diets or RNA reagents had to be administered to the corresponding groups. Fluorescence images were acquired using identical microscope settings across experimental groups and were coded before quantitative analysis so that the investigator performing fluorescence quantification was unaware of treatment identity.

### 2.3. Spore Inoculation

Larvae were reared and inoculated with *A. apis* spores as described in the literature [30]. Briefly, the larval diet was prepared, preheated to 35 °C, and distributed into 6-well culture plates. Two-day-old larvae were transferred into the plates using a grafting needle. After 24 h, the larvae were moved to 48-well plates (one larva per well) and incubated at 35 ± 0.5 °C with 90% relative humidity. Purified *A. apis* spores were serially diluted and mixed with the larval diet to a final concentration of 2 × 10^7^ spores/mL. On day 3, each larva in the treatment group received 50 µL of the spore-containing diet, corresponding to an administered dose of 1 × 10^6^ spores per larva (0 dpi), while control larvae received an equal volume of spore-free diet. Spores were administered only at this initial feeding. Thereafter, all larvae received 50 µL of fresh spore-free diet every 24 h until day 6.

Larval guts were collected on days 4, 5, and 6, corresponding to 1, 2, and 3 dpi, respectively. Nine larvae were assigned to each experimental group at each sampling time, and all nine larvae were successfully collected. For each group at each dpi, gut tissues from three larvae originating from a single source colony were pooled to form one biological replicate, yielding three pooled biological replicates and nine larvae per group at each sampling time. Samples collected at 1, 2, and 3 dpi were obtained from different larvae because gut collection required destructive dissection; therefore, the observations at different dpi did not represent repeated measurements of the same individuals. The pooled gut samples were placed in RNase-free tubes, immediately frozen in liquid nitrogen, and stored at −80 °C until subsequent analysis.

### 2.4. Dual-Luciferase Reporter Gene Assay

Mimics for novel-miR0032-5p (M-miR0032-5p) and corresponding negative control mimic (M-NC) were synthesized by Shanghai Genepharma Co., Ltd. (Shanghai, China). Potential binding sites between lncRNA1386.1 and novel-miR0032-5p and between *CAT* and novel-miR0032-5p were predicted utilizing the RNAhybrid software (v.2.1.2). Primers were designed to amplify these binding sites, which were further cloned into the pmirGLO vector. The resulting recombinant plasmids, pmirGLO-lnc1386.1-wt and pmirGLO-CAT-wt, were used to generate the mutated plasmids pmirGLO-lnc1386.1-mut and pmirGLO-CAT-mut. All these recombinant plasmids were verified by Sanger sequencing, followed by plasmid extraction with an endotoxin-free plasmid extraction kit (Beijing TransGen Biotech Co., Ltd, Beijing, China).

HEK-293T cells were seeded into 48-well plates and cultured at 37 °C until they reached 90–95% confluence. This cell-density range was selected according to the manufacturer’s instructions for the Hieff Trans™ Liposomal Transfection Reagent (Yeasen Biotechnology Co., Ltd., Shanghai, China). The same confluence range was used for all experimental groups.

Co-transfection was performed using Hieff Trans™ Liposomal Transfection Reagent. Each well received 100 ng of the corresponding pmirGLO reporter plasmid, 10 pmol of M-miR0032-5p or M-NC, and 0.5 μL of transfection reagent. Eight transfection groups were established: lncRNA1386.1-wt + M-miR0032-5p, lncRNA1386.1-wt + M-NC, lncRNA1386.1-mut + M-miR0032-5p, lncRNA1386.1-mut + M-NC, CAT-wt + M-miR0032-5p, CAT-wt + M-NC, CAT-mut + M-miR0032-5p, and CAT-mut + M-NC. Three independent transfection experiments were conducted on separate occasions using independently prepared cell cultures and transfection mixtures. Six technical replicate wells were included for each group in each independent experiment. The values obtained from the six technical replicate wells were averaged to generate one value for each group in each experiment. After 24 h of transfection, cells were harvested, lysed, and centrifuged at 10,000 rpm for 1 min. Subsequently, 20 µL of the supernatant was collected, and firefly and Renilla luciferase activities were measured using a dual-luciferase reporter assay kit (Yeasen Biotechnology (Shanghai) Co., Ltd., Shanghai, China) in conjunction with a dual-luciferase assay system (Promega Corporation, Madison, WI, USA), following the manufacturers’ instructions. Relative luciferase activity was calculated as the ratio of firefly luciferase activity to Renilla luciferase activity.

### 2.5. lncRNA1386.1 Silencing

The designed siRNAs targeting lncRNA1386.1 (si-lncRNA1386.1) and corresponding negative control siRNAs (si-scramble) were synthesized by Shanghai Sangon Biotech Co., Ltd. (Shanghai, China). For each group, nine 3-day-old larvae were used at each sampling time. Larvae in the si-lncRNA1386.1 group were fed 50 µL of diet containing spores (1 × 10^6^ spores/larva) and si-lncRNA1386.1 (2 μg/larva). After 24 h, the larvae were provided with diet containing only si-lncRNA1386.1 until 6-day-old. Larvae in the si-scramble group were fed 50 µL of diet containing the same dose of *A. apis* spores and si-scramble (2 μg/larva). Using our previously established method [30], gut tissues were collected from 4-, 5-, and 6-day-old larvae, corresponding to 1, 2, and 3 dpi, respectively. All nine larvae in each group were successfully collected at every sampling time. Gut tissues from three larvae originating from a single source colony were pooled to form one biological replicate, yielding three pooled biological replicates per group at each dpi. The pooled gut samples were frozen in liquid nitrogen and stored at −80 °C until analysis.

### 2.6. Overexpression and Inhibition of novel-miR0032-5p

Mimics (M-miR0032-5p) and inhibitors (I-miR0032-5p), along with negative control mimics (M-NC) and inhibitors (I-NC), were synthesized by Shanghai Genepharma Co., Ltd. (Shanghai, China) (Table S1). *A. apis*-infected larvae (3 days old) were fed with 50 μL of spore-containing diet and M-miR0032-5p or I-miR0032-5p (60 pmol/g). After 24 h, larvae were supplemented with diet containing only the corresponding mimic or inhibitor until day 6. Control larvae received M-NC or I-NC. Larval guts were collected on days 4, 5, and 6 (1, 2, and 3 dpi, respectively). Nine larvae were assigned to each group at each sampling time. At each sampling time, gut tissues from three larvae originating from a single source colony were pooled to form one biological replicate, yielding three pooled biological replicates and nine larvae per group at each dpi. The pooled gut samples were frozen in liquid nitrogen and stored for further analysis.

### 2.7. Quantitative Real-Time PCR (qRT-PCR)

Total RNA was extracted from gut samples of 4-, 5-, and 6-day-old larvae, with three biological replicates included for each group at each time point. Each replicate consisted of gut tissues pooled from three larvae from the same colony, as described in Section 2.2. Reverse transcription was performed using a stem-loop RT primer for novel-miR0032-5p, random primers for *U*6 and 5.8*S rRNA*, and oligo(dT) primers for lncRNA1386.1 and the protein-coding transcripts. Following the manufacturer’s instruction for the Hifair^®^ qPCR SYBR Green Master Mix (Yeasen Biotechnology Co., Ltd., Shanghai, China), qRT-PCR was conducted on a QuantStudio 3 Real-Time PCR system (Applied Biosystems, Foster City, CA, USA). The *U*6 gene (GenBank accession number: LOC725641) was used as the internal reference for novel-miR0032-5p, while the *actin* gene (GenBank accession number: LOC107999330) served as the internal reference for lncRNA1386.1, *CAT*, *Relish* (GenBank accession number: LOC107995490), and *Dorsal*1 (GenBank accession number: LOC107996080). For the *A. apis Chit*3 (GenBank accession number: KZZ95064.1) and *STE*11*-like* (GenBank accession number: EF156415.1), the 5.8*S rRNA* gene (GenBank accession number: U68313.1) was used as the internal reference. The stability of *actin*, *U*6, and 5.8*S rRNA* was evaluated from their raw Ct values across treatments and sampling times. Their Ct ranges and coefficients of variation are reported in Table S2, and no significant treatment- or time-associated variation was detected.

The sequences of the primers used in this study are provided in Table S1. The qPCR reaction system (20 μL) included: 10 μL of Hifair^®^ qPCR SYBR Green, 1 μL each of the forward and reverse primers (2.5 μM each), 1 μL of cDNA template, and 7 μL of DEPC water. The reaction conditions were set as follows: 95 °C for 3 min for pre-denaturation, followed by 95 °C for 10 s for denaturation, 60 °C for 30 s for annealing and extension, for a total of 42 cycles. Melting-curve analysis was performed after amplification, and all primer pairs produced a single specific peak. Amplification efficiencies ranged from 96.6% to 101.1%, with R^2^ values ranging from 0.9962 to 0.9992 (Table S2).

Each biological replicate was run in triplicate. Ct values from valid technical wells were averaged before relative expression was calculated, and technical replicates were not treated as independent samples. Relative expression levels were determined using the 2^−ΔΔCt^ method after confirming comparable amplification efficiencies between the target and reference assays.

### 2.8. DHE Staining of Larval Gut Tissue

Larval guts were collected from 4-, 5-, and 6-day-old larvae as described above. The assay included the uninoculated, *A. apis*-inoculated, *A. apis* + si-lncRNA1386.1, *A. apis* + si-scramble, *A. apis* + M-miR0032-5p, *A. apis* + M-NC, *A. apis* + I-miR0032-5p, and *A. apis* + I-NC groups. DHE-derived fluorescence was interpreted as a superoxide-associated signal rather than a direct measure of total reactive oxygen species.

Freshly dissected guts were rinsed once with 1× PBS, incubated with 5 μM dihydroethidium (DHE; Yeasen Biotechnology (Shanghai) Co., Ltd., Shanghai, China) for 20 min at room temperature in the dark, washed three times with 1× PBS, and imaged immediately without fixation. Images were acquired using a Nikon ECLIPSE Ts2-FL inverted fluorescence microscope (Nikon Corporation, Tokyo, Japan) equipped with a 20× objective and the C-LED525 fluorescence channel. The excitation and emission ranges were approximately 500–550 and 568–626 nm, respectively. Exposure time and detector gain were fixed at 558.33 ms and 1.00, and all acquisition settings were identical across groups.

Three pooled biological replicates were analyzed per group at each time point (*n* = 3). Each replicate comprised three guts originating from a single source colony, with three predefined non-overlapping fields acquired from each gut. Field selection and region-of-interest delineation were performed using transmitted-light images without reference to DHE fluorescence intensity. Fluorescence was quantified from the original images using ImageJ (version 1.54j; National Institutes of Health, Bethesda, MD, USA) [32]. The mean intensity of three tissue-free background regions was subtracted from the mean intensity of the gut-tissue region of interest. Values from the three fields were averaged per gut, and values from the three guts were subsequently averaged to obtain one biological replicate. Individual fields and guts were not treated as independent observations. Images were coded before quantification to blind the investigator to treatment allocation, and any brightness or contrast adjustments used for presentation were applied equally and were not used for quantitative analysis [32].

### 2.9. Measurement of CAT Protein Concentration

CAT protein concentration in larval gut homogenates was measured using an Insect Catalase (CAT) ELISA Kit (Cat. No. YJ332054; Shanghai Yuanju Bio-Tech Co., Ltd., Shanghai, China) according to the manufacturer’s instructions. Gut samples were collected from 4-, 5-, and 6-day-old larvae in the *A. apis* + si-lncRNA1386.1, *A. apis* + si-scramble, *A. apis* + M-miR0032-5p, *A. apis* + M-NC, *A. apis* + I-miR0032-5p, and *A. apis* + I-NC groups. Three pooled biological replicates were analyzed per group at each time point, with each replicate comprising three pooled larval guts, as described in Section 2.2. The pooled guts were homogenized in physiological saline and centrifuged at 3000× *g* for 10 min at 4 °C. The resulting supernatant was analyzed using a one-step double-antibody sandwich ELISA. Briefly, standards or samples were added to wells pre-coated with an anti-insect CAT capture antibody together with an HRP-conjugated detection antibody and incubated at 37 °C for 60 min. After washing, TMB substrate was added, and the plate was incubated at 37 °C for 15 min in the dark. The reaction was terminated with stop solution, and absorbance was measured at 450 nm using a Varioskan LUX multimode microplate reader (Thermo Fisher Scientific, Waltham, MA, USA). The standard curve ranged from 100 to 10,000 pg/mL. CAT protein concentrations were calculated after blank subtraction and corrected for the dilution factor.

### 2.10. Measurement of Survival Rate and Chalkbrood Incidence

Using the procedures described above, 3-day-old larvae were inoculated with *A. apis* spores and subsequently treated once daily with si-lncRNA1386.1, si-scramble, novel-miR0032-5p mimic, novel-miR0032-5p inhibitor, M-NC, or I-NC until day 6. Visible chalkbrood manifestation was defined as the first observation of external fungal mycelial growth on the body surface of a dead larva. Dead larvae were retained in their individual wells and examined daily until visible mycelial growth was observed or until the end of the scheduled observation period. Because death preceded rather than precluded the appearance of external mycelia, death was regarded as an intermediate state and was not treated as a competing event or censoring event.

Each colony contributed 16 larvae to each treatment group. Daily cumulative percentages were converted to numbers of larvae showing visible mycelial growth, with an increment of 6.25% corresponding to one larva. The first day on which visible mycelial growth was recorded was used as the event time. Larvae without visible mycelial growth by the end of the corresponding observation period were right censored at the final observation day.

### 2.11. Statistical Analysis

Statistical analysis of the data was performed using SPSS Statistics 21.0 software (IBM Corp., Armonk, NY, USA) and GraphPad Prism 10.0 software (GraphPad Software, Boston, MA, USA). The relative expression levels of novel-miR0032-5p, lncRNA1386.1, *CAT*, *Relish*, *Dorsal*1, *Chit*3, and *STE*11*-like* were calculated using the 2^−ΔΔCt^ method [33].

Time-course data, including relative transcript abundance, DHE fluorescence intensity, and CAT protein concentration, were analyzed separately for each experiment using ordinary two-way ANOVA with treatment and dpi as fixed factors, including the treatment × dpi interaction. The lncRNA1386.1-silencing, novel-miR0032-5p-mimic, and novel-miR0032-5p-inhibitor experiments were analyzed separately because each experiment had its own corresponding negative control. Samples collected at different dpi were obtained from different larvae and were therefore analyzed as independent observations rather than repeated measurements. Šídák-adjusted pairwise comparisons were performed between each treatment and its corresponding negative control at each dpi. Model residuals were assessed for normality and homogeneity of variance.

Dual-luciferase reporter data were analyzed using two-way ANOVA with reporter construct and miRNA treatment as fixed factors, followed by Šídák-adjusted pairwise comparisons.

Larval survival was analyzed using Kaplan–Meier survival analysis. The lncRNA1386.1-silencing, novel-miR0032-5p-overexpression, and novel-miR0032-5p-inhibition experiments were analyzed separately because each experiment had its own matched negative control and observation period. Time to first visible external mycelial growth was analyzed using Cox proportional hazards regression stratified by colony, with treatment included as the explanatory variable. The Efron method was used to account for tied events. Hazard ratios (HRs), 95% confidence intervals (CIs), and *p*-values were reported.

A two-sided *p* value < 0.05 was considered statistically significant. Statistical significance is indicated as follows: ns, *p* > 0.05; *, *p* < 0.05; **, *p* < 0.01; ***, *p* < 0.001; and ****, *p* < 0.0001. Data are presented as the mean ± standard deviation (SD), with individual biological replicate values shown where applicable.

## 3. Results

### 3.1. Expression Profiles of lncRNA1386.1, novel-miR0032-5p, and CAT in the A. apis Infection Process

**Figure 1:**
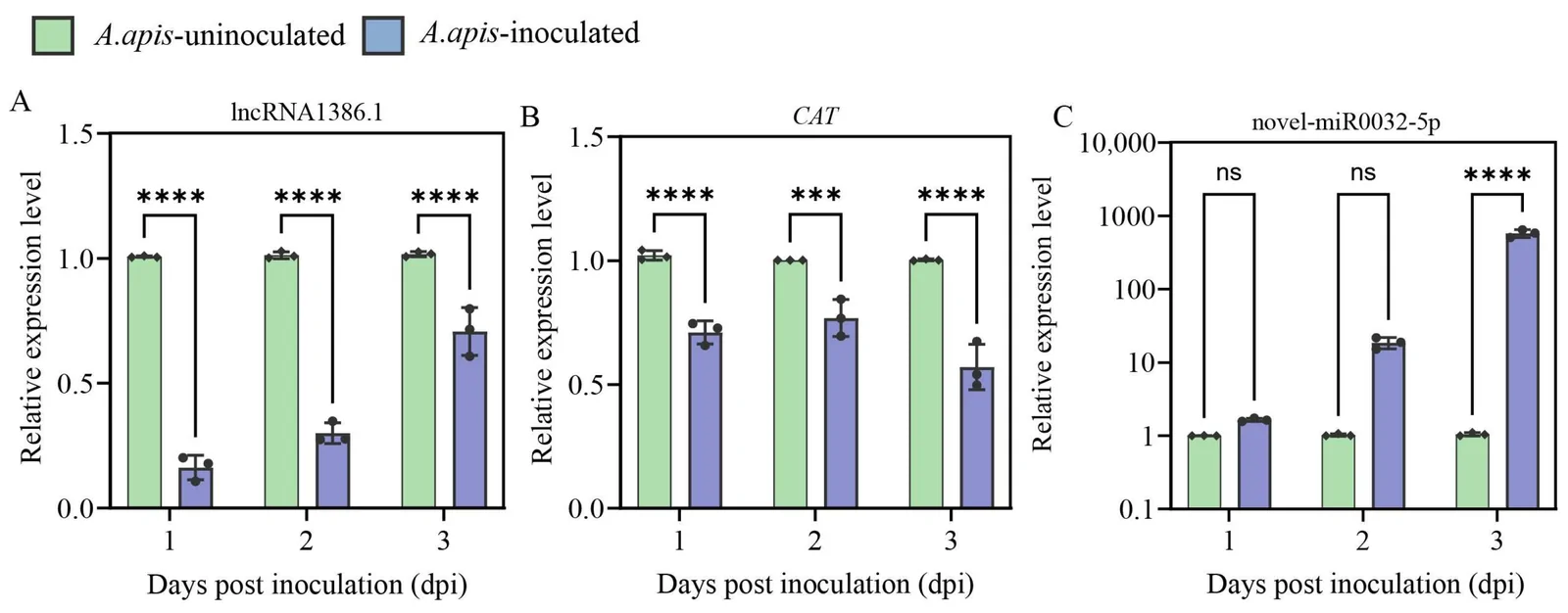
The expression profiles of lncRNA1386.1, novel-miR0032-5p, and *CAT* in the guts of *A. cerana* worker larvae inoculated with *A. apis*. (**A**) lncRNA1386.1. (**B**) *CAT*. (**C**) novel-miR0032-5p. Bars represent the mean ± SD, and individual points represent pooled biological replicates, each comprising three larval guts (*n* = 3 per group at each dpi). Technical replicates were averaged before statistical analysis. Data were analyzed using ordinary two-way ANOVA with treatment and dpi as fixed factors, followed by Šídák-adjusted pairwise comparisons between the *A. apis*-inoculated and uninoculated groups at each dpi. The y-axis in panel (**C**) is shown on a logarithmic scale. Brackets and symbols indicate the results of the corresponding Šídák-adjusted comparisons. ns, *p* > 0.05; ***, *p* < 0.001; ****, *p* < 0.0001.

The results showed significant treatment × dpi interactions for lncRNA1386.1 (*F*_2,12_ = 51.18, *p* < 0.0001), *CAT* (*F*_2,12_ = 5.472, *p* = 0.0205), and novel-miR0032-5p (*F*_2,12_ = 197.4, *p* < 0.0001). lncRNA1386.1 transcript abundance was significantly lower in the *A. apis*-inoculated group at 1, 2, and 3 dpi (*p* < 0.0001) (Figure 1A). *CAT* transcript abundance was also significantly reduced at 1 dpi (*p* < 0.0001), 2 dpi (*p* < 0.001), and 3 dpi (*p* < 0.0001) (Figure 1B). In contrast, novel-miR0032-5p transcript abundance did not differ significantly between groups at 1 or 2 dpi (*p* > 0.05), but was significantly elevated in the inoculated group at 3 dpi (*p* < 0.0001) (Figure 1C). Thus, *A. apis* infection was associated with sustained reductions in lncRNA1386.1 and *CAT* expression, whereas novel-miR0032-5p upregulation was mainly observed at 3 dpi.

### 3.2. Predicted-Site-Dependent Reporter Responses of CAT and lncRNA1386.1 to novel-miR0032-5p

**Figure 2:**
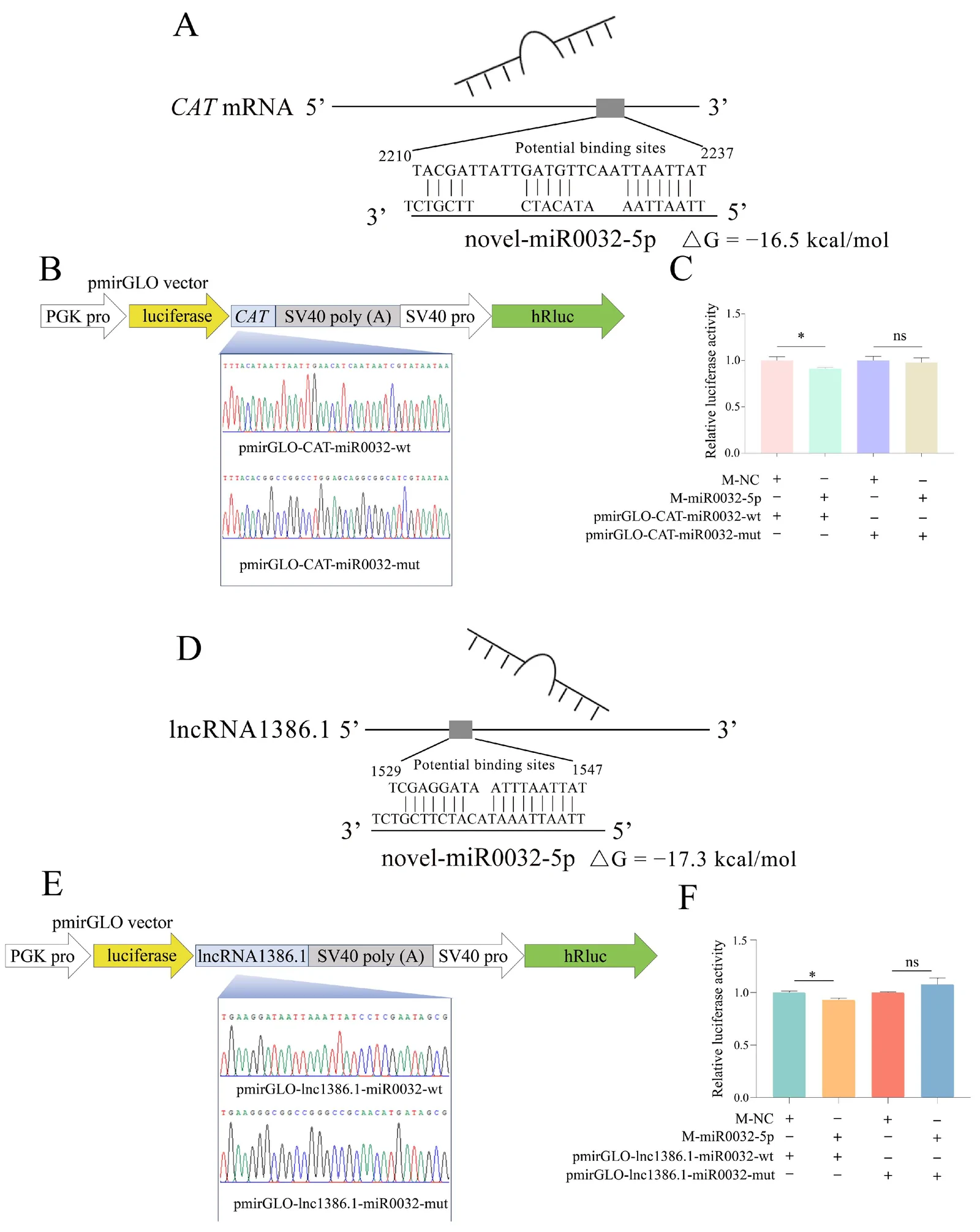
Dual-luciferase reporter gene assay for the binding relationships among lncRNA1386.1, novel-miR0032-5p, and *CAT*. (**A**,**D**) Schematic diagrams of the potential targeting sites between lncRNA1386.1 and novel-miR0032-5p and between novel-miR0032-5p and *CAT*. (**B**,**E**) pmirGLO vector construction model for dual-luciferase reporter gene assay and peak diagrams of Sanger sequencing of the binding sites and mutated binding sites. (**C**,**F**) Relative luciferase activity of HEK-293T cells co-transfected with mimic and recombinant plasmids. Data were analyzed by two-way ANOVA followed by Šídák-adjusted comparisons. *, *p* < 0.05; ns, *p* > 0.05.

The potential binding sites between novel-miR0032-5p and *CAT* mRNA and between lncRNA1386.1 and novel-miR0032-5p were presented in Figure 2A,D. Recombinant plasmids for the dual-luciferase assay were successfully constructed, including pmirGLO-CAT-miR0032-wt, pmirGLO-CAT-miR0032-mut, pmirGLO-lnc1386.1-miR0032-wt, and pmirGLO-lnc1386.1-miR0032-mut (Figure 2B,E). Compared with cells co-transfected with M-NC and pmirGLO-CAT-miR0032-wt, cells co-transfected with M-miR0032-5p and pmirGLO-CAT-miR0032-wt showed significantly reduced relative luciferase activity (*p* < 0.05). Similarly, a significant decrease (*p* < 0.05) was observed following co-transfection of cells with M-miR0032-5p and pmirGLO-lnc1386.1-miR0032-wt. However, this reduction in luciferase activity was not observed when the predicted binding sites in *CAT* or lncRNA1386.1 were mutated (Figure 2C,F).

### 3.3. lncRNA1386.1 Silencing Affected the Expression of novel-miR0032-5p and CAT in the Larval Guts During A. apis Infection

**Figure 3:**
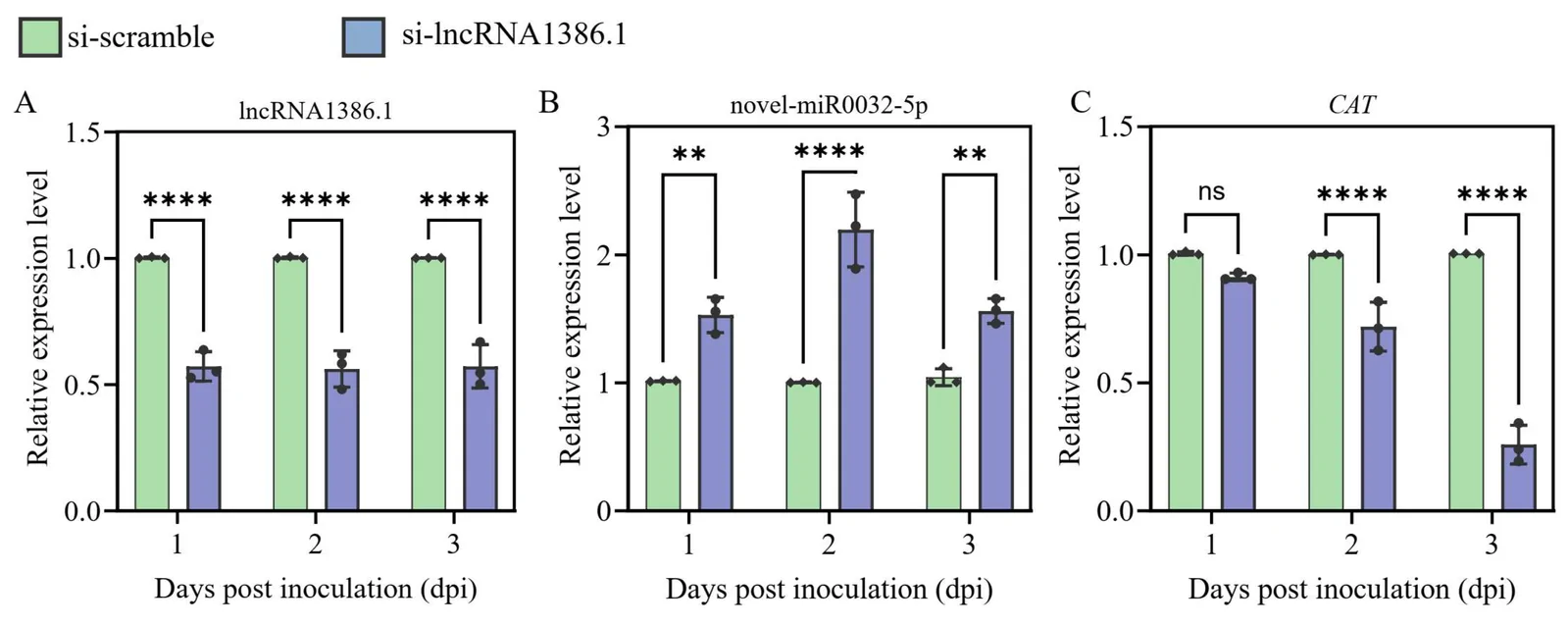
Effects of lncRNA1386.1 silencing on lncRNA1386.1, novel-miR0032-5p, and *CAT* transcript abundance in the guts of *Apis cerana* worker larvae inoculated with *Ascosphaera apis*. (**A**) lncRNA1386.1. (**B**) novel-miR0032-5p. (**C**) *CAT*. Bars represent the mean ± SD, and individual points represent biological replicates (*n* = 3 per group at each dpi), with each replicate comprising three pooled larval guts. Technical replicates were averaged before statistical analysis. Data were analyzed using ordinary two-way ANOVA with treatment and dpi as fixed factors, followed by Šídák-adjusted pairwise comparisons between the si-lncRNA1386.1 and si-scramble groups at each dpi. Brackets and symbols indicate the results of the corresponding Šídák-adjusted comparisons. ns, *p* > 0.05; **, *p* < 0.01; ****, *p* < 0.0001.

The results showed no significant treatment × dpi interaction for lncRNA1386.1 transcript abundance (*F*_2,12_ = 0.025, *p* > 0.05), but identified a significant treatment effect (*F*_1,12_ = 318.9, *p* < 0.0001). The results confirmed significant lncRNA1386.1 silencing at 1, 2, and 3 dpi (*p* < 0.0001) (Figure 3A). Significant treatment × dpi interactions were detected for novel-miR0032-5p (*F*_2,12_ = 11.68, *p* < 0.01) and *CAT* (*F*_2,12_ = 67.45, *p* < 0.0001). novel-miR0032-5p transcript abundance was significantly increased at 1 dpi (*p* < 0.01), 2 dpi (*p* < 0.0001), and 3 dpi (*p* < 0.01) following lncRNA1386.1 silencing (Figure 3B). *CAT* transcript abundance was not significantly altered at 1 dpi (*p* > 0.05), but was significantly reduced at 2 and 3 dpi (*p* < 0.0001) (Figure 3C).

### 3.4. Overexpression and Inhibition of novel-miR0032-5p Affected the Expression of lncRNA1386.1 and CAT in the A. apis-Infected Larval Guts

**Figure 4:**
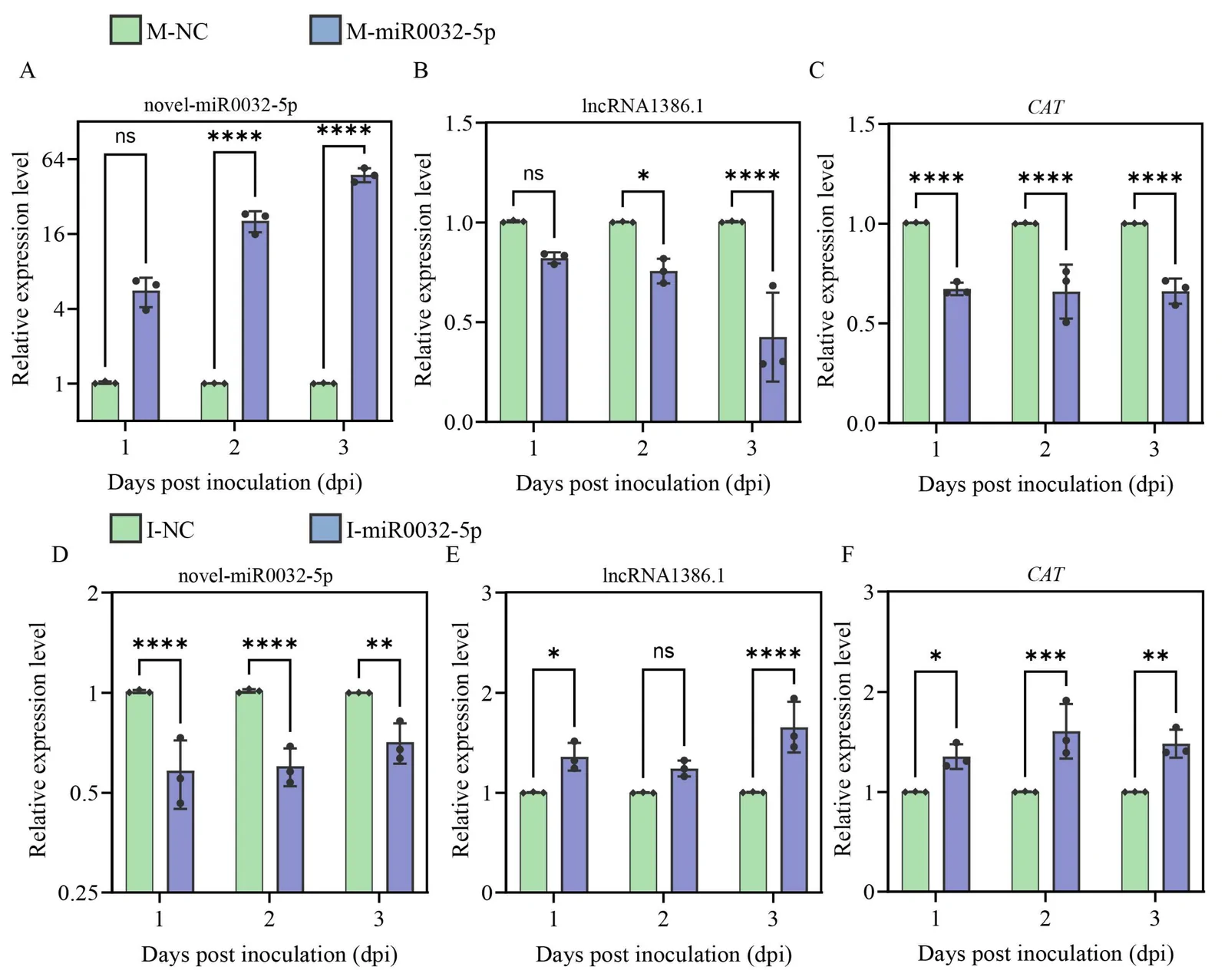
Effects of novel-miR0032-5p overexpression and inhibition on novel-miR0032-5p, lncRNA1386.1, and *CAT* transcript abundance in the guts of *Apis cerana* worker larvae inoculated with *Ascosphaera apis*. (**A**–**C**) Transcript abundance of novel-miR0032-5p, lncRNA1386.1, and *CAT*, respectively, following novel-miR0032-5p mimic administration. (**D**–**F**) Transcript abundance of novel-miR0032-5p, lncRNA1386.1, and *CAT*, respectively, following novel-miR0032-5p inhibitor administration. Bars represent the mean ± SD, and individual points represent biological replicates (*n* = 3 per group at each dpi), with each replicate comprising three pooled larval guts. Technical replicates were averaged before statistical analysis. Data were analyzed using ordinary two-way ANOVA with treatment and dpi as fixed factors, followed by Šídák-adjusted pairwise comparisons between each treatment and its corresponding negative control at each dpi. The y-axes in panels A and D are shown on logarithmic scales. Brackets and symbols indicate the results of the corresponding Šídák-adjusted comparisons. ns, *p* > 0.05; *, *p* < 0.05; **, *p* < 0.01; ***, *p* < 0.001; ****, *p* < 0.0001.

The results identified significant treatment × dpi interactions for novel-miR0032-5p (*F*_2,12_ = 73.36, *p* < 0.0001) and lncRNA1386.1 (*F*_2,12_ = 7.468, *p* < 0.01) transcript abundance following novel-miR0032-5p mimic administration. The results showed that novel-miR0032-5p transcript abundance was not significantly altered at 1 dpi (*p* > 0.05), but was significantly increased at 2 and 3 dpi (*p* < 0.0001) (Figure 4A). lncRNA1386.1 transcript abundance was not significantly altered at 1 dpi (*p* > 0.05), but was significantly reduced at 2 dpi (*p* < 0.05) and 3 dpi (*p* < 0.0001) (Figure 4B). No significant treatment × dpi interaction was detected for *CAT* transcript abundance (*F*_2,12_ = 0.010, *p* > 0.05), whereas the main effect of treatment was significant (*F*_1,12_ = 132.4, *p* < 0.0001). *CAT* transcript abundance was significantly lower in the M-miR0032-5p group at 1, 2, and 3 dpi (*p* < 0.0001) (Figure 4C).

In the inhibitor experiment, no significant treatment × dpi interaction was detected for novel-miR0032-5p transcript abundance (*F*_2,12_ = 1.454, *p* > 0.05), whereas the main effect of treatment was significant (*F*_1,12_ = 109.9, *p* < 0.0001). novel-miR0032-5p transcript abundance was significantly lower in the I-miR0032-5p group at all examined dpi (1 and 2 dpi, *p* < 0.0001; 3 dpi, *p* < 0.01) (Figure 4D). A significant treatment × dpi interaction was detected for lncRNA1386.1 transcript abundance (*F*_2,12_ = 4.472, *p* < 0.05). lncRNA1386.1 transcript abundance was significantly higher following novel-miR0032-5p inhibition at 1 dpi (*p* < 0.05) and 3 dpi (*p* < 0.0001), but not at 2 dpi (*p* > 0.05) (Figure 4E). No significant treatment × dpi interaction was detected for *CAT* transcript abundance (*F*_2,12_ = 1.301, *p* > 0.05), whereas the main effect of treatment was significant (*F*_1,12_ = 57.23, *p* < 0.0001). *CAT* transcript abundance was significantly higher in the I-miR0032-5p group at 1 dpi (*p* < 0.05), 2 dpi (*p* < 0.001), and 3 dpi (*p* < 0.05) (Figure 4F).

### 3.5. Changes in Host Immune-Related and Selected Fungal Transcript Abundance Following lncRNA1386.1 Silencing and novel-miR0032-5p Overexpression or Inhibition

The results showed significant treatment × dpi interactions for *Dorsal*1 following lncRNA1386.1 silencing (*F*_2,12_ = 7.595, *p* < 0.01), *Relish* following lncRNA1386.1 silencing (*F*_2,12_ = 14.15, *p* < 0.001), *Chit*3 following novel-miR0032-5p overexpression (*F*_2,12_ = 21.55, *p* < 0.0001) or inhibition (*F*_2,12_ = 8.008, *p* < 0.01), and *STE11-like* following novel-miR0032-5p inhibition (*F*_2,12_ = 38.01, *p* < 0.0001). The main effects of treatment were significant in all analyses.

**Figure 5:**
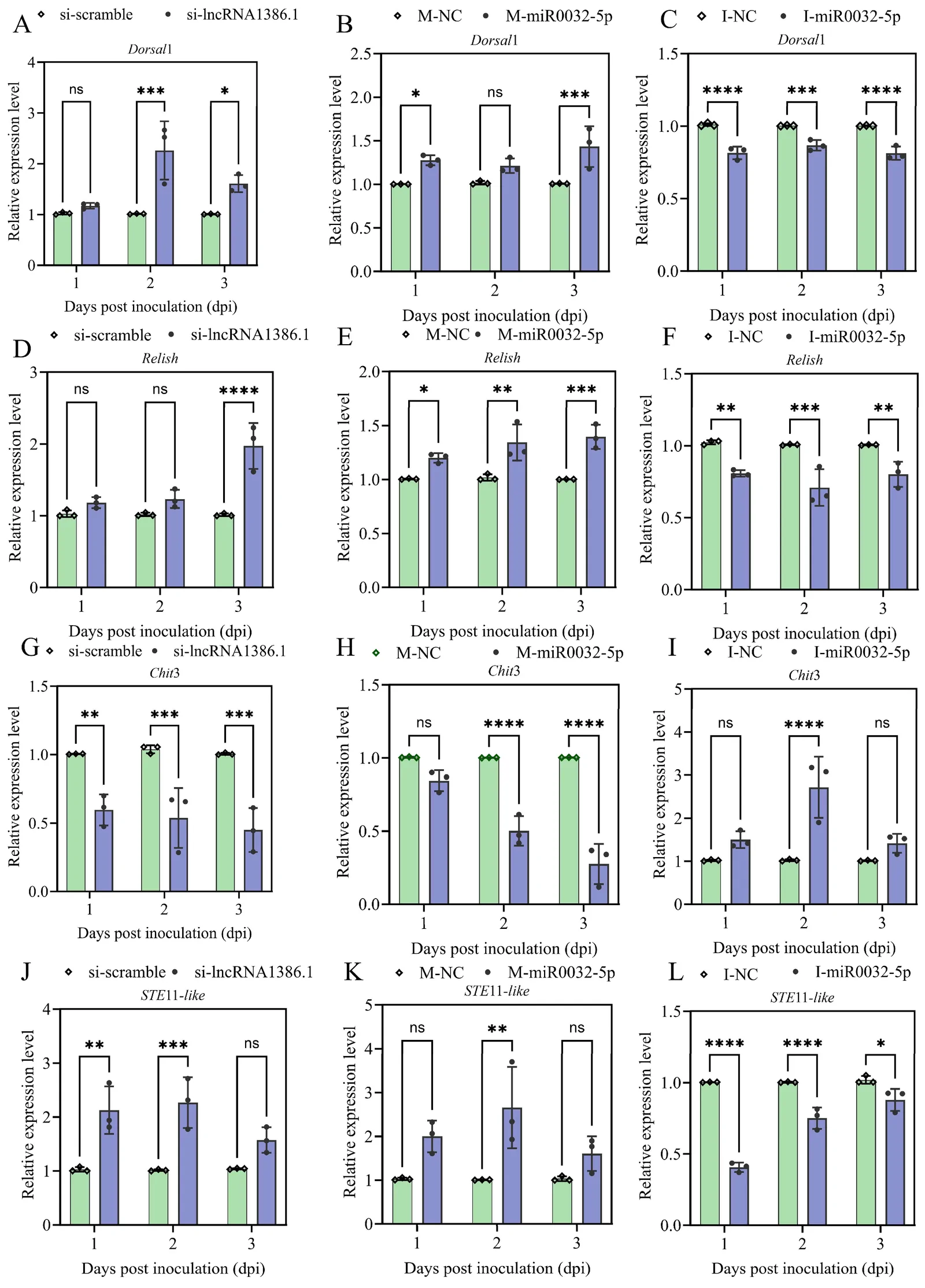
Effects of lncRNA1386.1 silencing and novel-miR0032-5p overexpression and inhibition on host immune-related and selected fungal transcript abundance in the guts of *A. cerana* worker larvae. (**A**–**C**) *Dorsal*1 transcript abundance following lncRNA1386.1 silencing, novel-miR0032-5p mimic administration, and novel-miR0032-5p inhibitor administration, respectively. (**D**–**F**) *Relish* transcript abundance following the corresponding treatments. (**G**–**I**) *Chit*3 transcript abundance following the corresponding treatments. (**J**–**L**) *STE*11-*like* transcript abundance following the corresponding treatments. Bars represent the mean ± SD, and individual points represent biological replicates (*n* = 3 per group at each dpi), with each replicate comprising three pooled larval guts. Technical replicates were averaged before statistical analysis. Data were analyzed using ordinary two-way ANOVA with treatment and dpi as fixed factors, followed by Šídák-adjusted pairwise comparisons between each treatment and its corresponding negative control at each dpi. Brackets and symbols indicate the results of the corresponding Šídák-adjusted comparisons. ns, *p* > 0.05; *, *p* < 0.05; **, *p* < 0.01; ***, *p* < 0.001; ****, *p* < 0.0001.

The results showed that lncRNA1386.1 silencing increased *Dorsal*1 transcript abundance at 2 and 3 dpi, but not at 1 dpi, and increased *Relish* transcript abundance only at 3 dpi (Figure 5A,D). novel-miR0032-5p overexpression increased *Dorsal*1 transcript abundance at 1 and 3 dpi and *Relish* transcript abundance at all examined dpi, whereas novel-miR0032-5p inhibition decreased both transcripts at all examined dpi (Figure 5B,C,E,F).

*Chit*3 transcript abundance was reduced following lncRNA1386.1 silencing at all examined dpi and following novel-miR0032-5p overexpression at 2 and 3 dpi, but not at 1 dpi. novel-miR0032-5p inhibition increased *Chit*3 transcript abundance only at 2 dpi (Figure 5G–I). *STE*11*-like* transcript abundance was increased following lncRNA1386.1 silencing at 1 and 2 dpi and following novel-miR0032-5p overexpression only at 2 dpi. Conversely, novel-miR0032-5p inhibition reduced *STE11-like* transcript abundance at all examined dpi (Figure 5J–L).

### 3.6. Increased DHE Fluorescence in Larval Guts Following A. apis Infection

**Figure 6:**
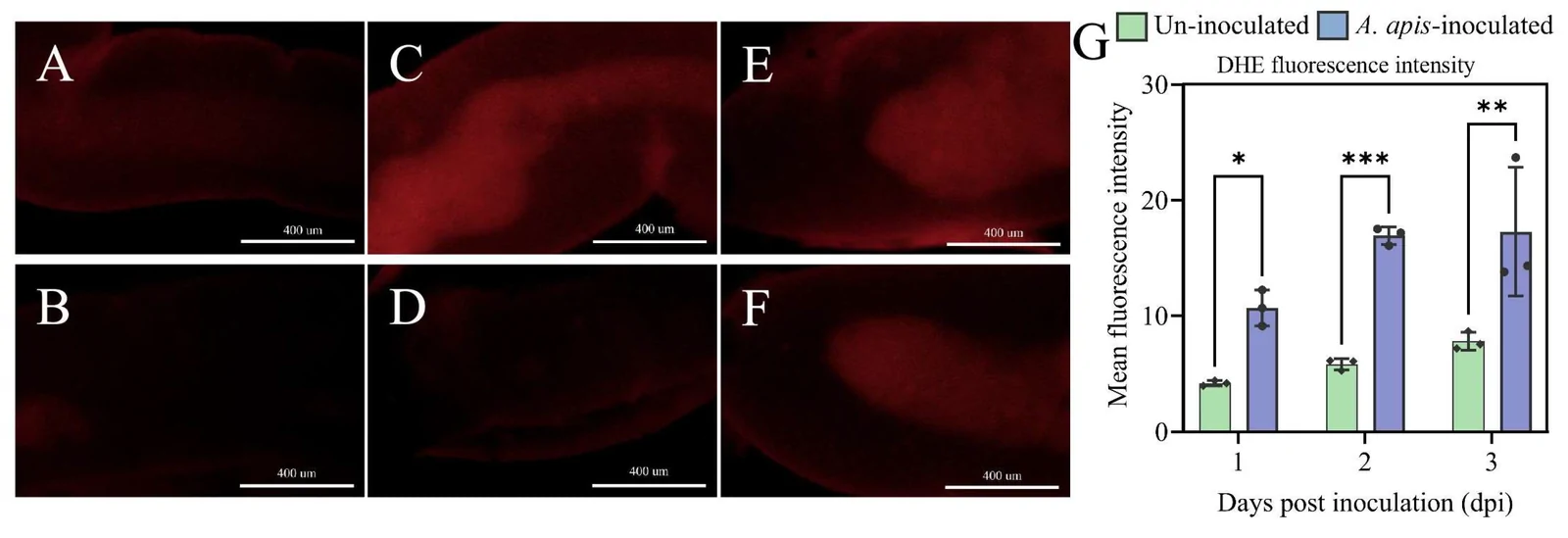
DHE fluorescence in the guts of *Apis cerana* worker larvae following *Ascosphaera apis* inoculation. Representative DHE fluorescence images of larval guts from the *A. apis*-inoculated groups at (**A**) 1 dpi, (**C**) 2 dpi, and (**E**) 3 dpi, and from the corresponding uninoculated groups at (**B**) 1 dpi, (**D**) 2 dpi, and (**F**) 3 dpi. (**G**) Quantification of mean DHE fluorescence intensity in inoculated and uninoculated larval guts at 1–3 dpi. Bars represent the mean ± SD, and individual points represent biological replicates (*n* = 3 per group at each dpi), with each replicate comprising three guts and three fields per gut. Measurements from fields and guts within the same colony were averaged before statistical analysis. Data were analyzed using ordinary two-way ANOVA with treatment and dpi as fixed factors, followed by Šídák-adjusted pairwise comparisons between the inoculated and uninoculated groups at each dpi. Brackets and symbols indicate the results of the corresponding Šídák-adjusted comparisons. *, *p* < 0.05; **, *p* < 0.01; ***, *p* < 0.001.

*A. apis*-inoculated larval guts exhibited higher DHE fluorescence than uninoculated controls (Figure 6A–F). No significant treatment × dpi interaction was detected (*F*_2,12_ = 1.401, *p* > 0.05), whereas the overall treatment effect was significant (*F*_1,12_ = 63.18, *p* < 0.0001). The results showed significantly higher DHE fluorescence intensity in the inoculated group at 1 dpi (*p* < 0.05), 2 dpi (*p* < 0.001), and 3 dpi (*p* < 0.01) (Figure 6G).

### 3.7. lncRNA1386.1 Silencing Was Associated with Elevated DHE Fluorescence in A. apis-Infected Larval Guts

**Figure 7:**
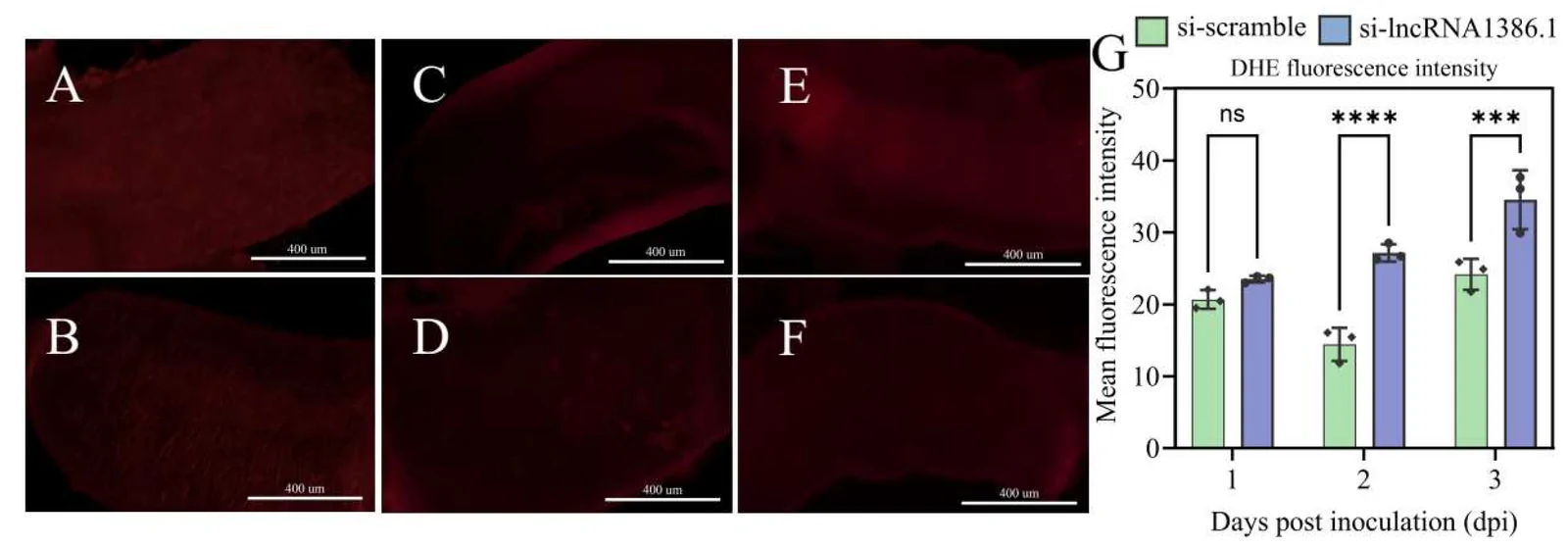
DHE fluorescence following lncRNA1386.1 silencing in the guts of *Apis cerana* worker larvae inoculated with *Ascosphaera apis*. Representative DHE fluorescence images of larval guts from the si-lncRNA1386.1 groups at (**A**) 1 dpi, (**C**) 2 dpi, and (**E**) 3 dpi, and from the corresponding si-scramble groups at (**B**) 1 dpi, (**D**) 2 dpi, and (**F**) 3 dpi. (**G**) Quantification of mean DHE fluorescence intensity in the si-lncRNA1386.1 and si-scramble groups at 1–3 dpi. Bars represent the mean ± SD, and individual points represent biological replicates (*n* = 3 per group at each dpi), with each replicate comprising three guts and three fields per gut. Measurements from fields and guts within the same colony were averaged before statistical analysis. Data were analyzed using ordinary two-way ANOVA with treatment and dpi as fixed factors, followed by Šídák-adjusted pairwise comparisons between the si-lncRNA1386.1 and si-scramble groups at each dpi. Brackets and symbols indicate the results of the corresponding Šídák-adjusted comparisons. ns, *p* > 0.05; ***, *p* < 0.001; ****, *p* < 0.0001.

A significant treatment × dpi interaction was detected for DHE fluorescence intensity following lncRNA1386.1 silencing (*F*_2,12_ = 7.980, *p* < 0.01). DHE fluorescence did not differ significantly between the si-lncRNA1386.1 and si-scramble groups at 1 dpi (*p* > 0.05), but was significantly higher in the si-lncRNA1386.1 group at 2 dpi (*p* < 0.0001) and 3 dpi (*p* < 0.001) (Figure 7).

### 3.8. novel-miR0032-5p Overexpression or Inhibition Was Associated with Alterations in DHE Fluorescence in A. apis-Infected Larval Guts

**Figure 8:**
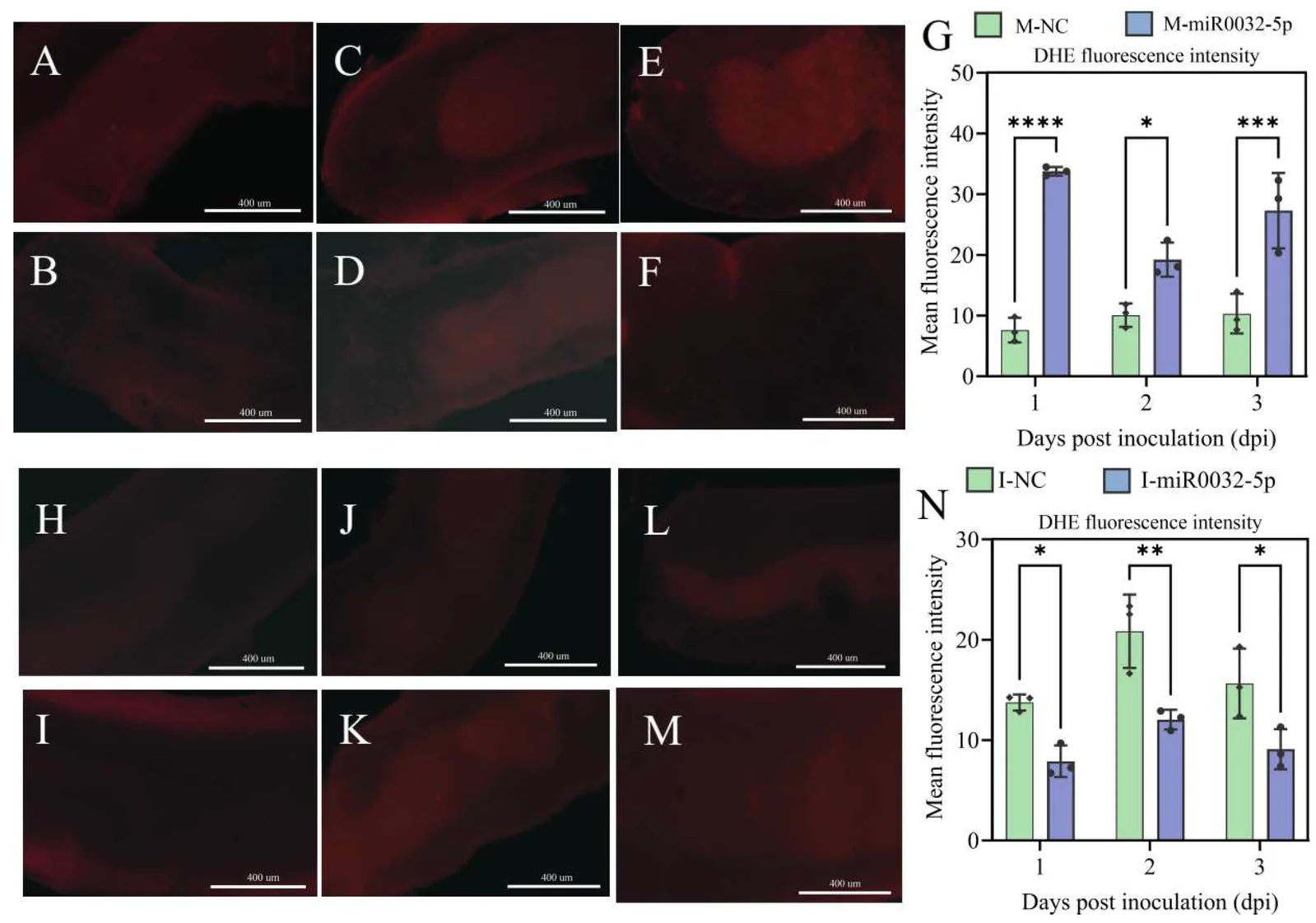
DHE fluorescence in the guts of *Apis cerana* worker larvae inoculated with *Ascosphaera apis* following novel-miR0032-5p mimic or inhibitor administration. Representative DHE fluorescence images of larval guts from the M-miR0032-5p groups at (**A**) 1 dpi, (**C**) 2 dpi, and (**E**) 3 dpi, and from the corresponding M-NC groups at (**B**) 1 dpi, (**D**) 2 dpi, and (**F**) 3 dpi. (**G**) Quantification of mean DHE fluorescence intensity in the M-miR0032-5p and M-NC groups at 1–3 dpi. Representative DHE fluorescence images of larval guts from the I-miR0032-5p groups at (**H**) 1 dpi, (**J**) 2 dpi, and (**L**) 3 dpi, and from the corresponding I-NC groups at (**I**) 1 dpi, (**K**) 2 dpi, and (**M**) 3 dpi. (**N**) Quantification of mean DHE fluorescence intensity in the I-miR0032-5p and I-NC groups at 1–3 dpi. Bars represent the mean ± SD, and individual points represent biological replicates (*n* = 3 per group at each dpi), with each replicate comprising three guts and three fields per gut. Measurements from fields and guts within the same colony were averaged before statistical analysis. Data were analyzed using ordinary two-way ANOVA with treatment and dpi as fixed factors, followed by Šídák-adjusted pairwise comparisons between each treatment and its corresponding negative control at each dpi. Brackets and symbols indicate the results of the corresponding Šídák-adjusted comparisons. *, *p* < 0.05; **, *p* < 0.01; ***, *p* < 0.001; ****, *p* < 0.0001.

In the mimic experiment, a significant treatment × dpi interaction was detected (*F*_2,12_ = 9.985, *p* < 0.01). DHE fluorescence intensity was significantly higher in the M-miR0032-5p group than in the M-NC group at 1 dpi (*p* < 0.0001), 2 dpi (*p* < 0.05), and 3 dpi (*p* < 0.001) (Figure 8A–G).

In the inhibitor experiment, no significant treatment × dpi interaction was detected (*F*_2,12_ = 0.637, *p* = 0.5461), whereas the overall treatment effect was significant (*F*_1,12_ = 40.22, *p* < 0.0001). DHE fluorescence intensity was significantly lower in the I-miR0032-5p group at 1 dpi (*p* < 0.05), 2 dpi (*p* < 0.01), and 3 dpi (*p* < 0.05) (Figure 8H–N).

### 3.9. Changes in CAT Protein Concentration Following lncRNA1386.1 Silencing and novel-miR0032-5p Overexpression or Inhibition

**Figure 9:**
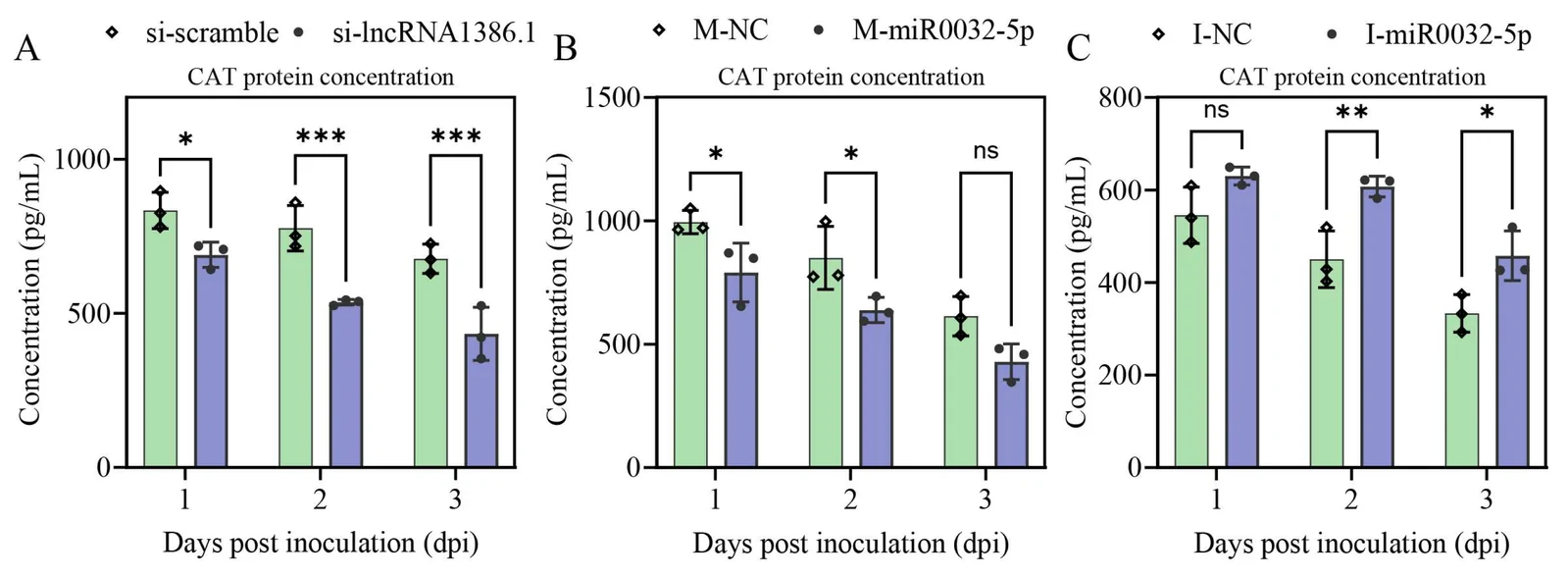
CAT protein concentration in the gut homogenates of *Apis cerana* worker larvae inoculated with *Ascosphaera apis* following lncRNA1386.1 silencing and novel-miR0032-5p overexpression or inhibition. CAT protein concentration following (**A**) lncRNA1386.1 silencing, (**B**) novel-miR0032-5p mimic administration, and (**C**) novel-miR0032-5p inhibitor administration. Bars represent the mean ± SD, and individual points represent biological replicates (*n* = 3 per group at each dpi), with each replicate comprising three pooled larval guts. The silencing, mimic, and inhibitor experiments were analyzed separately using ordinary two-way ANOVA with treatment and dpi as fixed factors, followed by Šídák-adjusted pairwise comparisons between each treatment and its corresponding negative control at each dpi. Brackets and symbols indicate the results of the corresponding Šídák-adjusted comparisons. ns, *p* > 0.05; *, *p* < 0.05; **, *p* < 0.01; ***, *p* < 0.001.

ELISA analysis showed no significant treatment × dpi interaction for CAT protein concentration following lncRNA1386.1 silencing (*F*_2,12_ = 1.412, *p* > 0.05), novel-miR0032-5p overexpression (*F*_2,12_ = 0.038, *p* > 0.05), or novel-miR0032-5p inhibition (*F*_2,12_ = 0.934, *p* > 0.05). Significant overall treatment effects were detected in all three experiments. The results showed that CAT protein concentration was significantly lower in the si-lncRNA1386.1 group than in the si-scramble group at 1 dpi (*p* < 0.05), 2 dpi (*p* < 0.001), and 3 dpi (*p* < 0.001) (Figure 9A). In the mimic experiment, CAT protein concentration was significantly lower in the M-miR0032-5p group at 1 dpi (*p* < 0.05) and 2 dpi (*p* < 0.05), but not at 3 dpi (*p* > 0.05) (Figure 9B). Conversely, novel-miR0032-5p inhibition significantly increased CAT protein concentration at 2 dpi (*p* < 0.01) and 3 dpi (*p* < 0.05), whereas no significant difference was detected at 1 dpi (*p* > 0.05) (Figure 9C).

### 3.10. Larval Survival and Chalkbrood-Associated Outcomes Following lncRNA1386.1 Silencing and novel-miR0032-5p Overexpression or Inhibition

**Figure 10:**
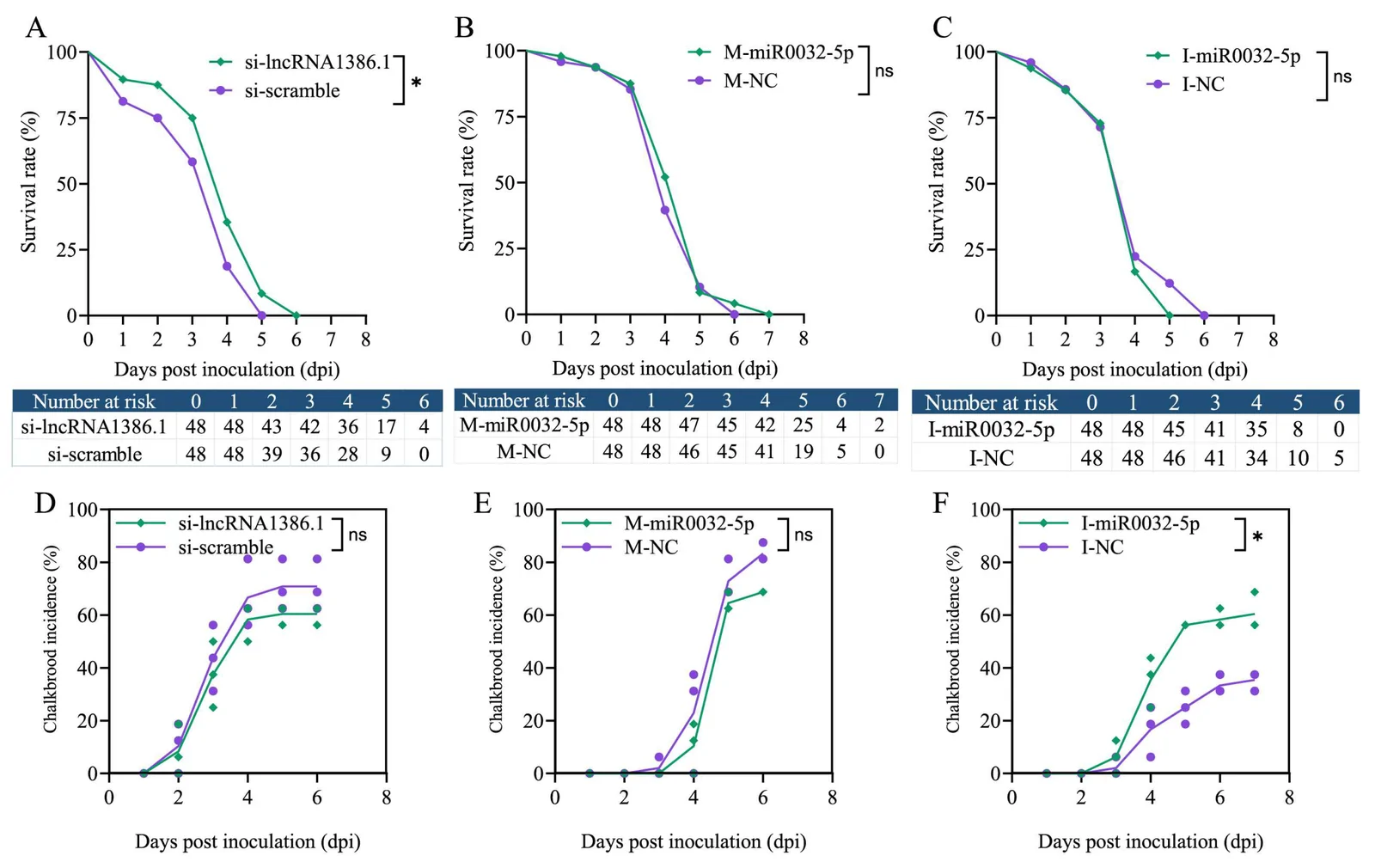
Larval survival and visible external mycelial growth following lncRNA1386.1 silencing or novel-miR0032-5p overexpression or inhibition during *Ascosphaera apis* infection. (**A**–**C**) Kaplan–Meier survival curves following (**A**) lncRNA1386.1 silencing, (**B**) novel-miR0032-5p mimic administration, and (**C**) novel-miR0032-5p inhibitor administration. Numbers at risk are shown below the curves, and treatment and corresponding control groups were compared using log-rank tests. (**D**–**F**) Cumulative proportions of larvae showing visible external mycelial growth following the corresponding treatments. Each point represents the cumulative proportion within one colony-derived biological replicate comprising 16 larvae, and lines connect the group means. Time to first visible external mycelial growth was analyzed using Cox proportional hazards models stratified by colony, with ties handled using the Efron method. Each group contained 48 larvae from three colonies, with 16 larvae contributed by each colony. Visible external mycelial growth on dead larvae was used as the predefined morphological endpoint. ns, *p* > 0.05; *, *p* < 0.05.

Kaplan–Meier analysis showed that larvae in the si-lncRNA1386.1 group had a significantly higher survival probability than those in the si-scramble group during the observation period (*p* < 0.05; Figure 10A). In contrast, no significant differences in larval survival were detected between the M-miR0032-5p and M-NC groups (*p* > 0.05; Figure 10B) or between the I-miR0032-5p and I-NC groups (*p* > 0.05; Figure 10C).

By 7 dpi, visible external mycelial growth was observed in 29 of 48 larvae (60.42%) in the si-lncRNA1386.1 group and 34 of 48 larvae (70.83%) in the si-scramble group. Colony-stratified Cox regression showed no significant difference in the time to first visible external mycelial growth between the two groups (HR = 0.76, 95% CI = 0.46–1.25, *p* > 0.05; Figure 10D). By 6 dpi, visible external mycelial growth was observed in 33 of 48 larvae (68.75%) in the M-miR0032-5p group and 40 of 48 larvae (83.33%) in the M-NC group. The time to first visible external mycelial growth did not differ significantly between the two groups (HR = 0.69, 95% CI = 0.43–1.10, *p* > 0.05; Figure 10E). By 8 dpi, visible external mycelial growth was observed in 29 of 48 larvae (60.42%) in the I-miR0032-5p group and 17 of 48 larvae (35.42%) in the I-NC group. novel-miR0032-5p inhibition was associated with a significantly higher hazard of first visible external mycelial growth than the control treatment (HR = 2.19, 95% CI = 1.20–4.00, *p* < 0.05; Figure 10F).

## 4. Discussion

### 4.1. An Infection-Responsive lncRNA1386.1/novel-miR0032-5p/CAT Regulatory Relationship Compatible with a ceRNA-like Axis in the Larval Gut

LncRNAs can participate in post-transcriptional regulation, including shared miRNA-responsive relationships that are compatible with competing endogenous RNA (ceRNA)-like models [26,27,34,35]. Here, lncRNA1386.1 and *CAT* display expression patterns opposite that of novel-miR0032-5p during *A. apis* infection (Figure 1A–C). In dual-luciferase reporter assays, M-miR0032-5p reduced the activity of reporters containing the predicted MRE in lncRNA1386.1 or *CAT*, whereas mutation of these sequences weakened or eliminated the response (Figure 2C,F). These results indicate that the predicted sequences can mediate novel-miR0032-5p-responsive reporter regulation in HEK-293T cells. In infected larvae, lncRNA1386.1 silencing and novel-miR0032-5p overexpression were accompanied by reduced *CAT* transcript abundance (Figure 3A–C), while novel-miR0032-5p inhibition produced an opposite expression pattern (Figure 4A–F). These coordinated changes are compatible with a shared miRNA-responsive regulatory relationship among lncRNA1386.1, novel-miR0032-5p, and *CAT*. Nevertheless, the present evidence does not establish that lncRNA1386.1 functions as an endogenous miRNA sponge or that competition for novel-miR0032-5p is the sole explanation for the observed expression changes. Altered RNA stability, indirect transcriptional regulation, and common upstream responses to infection or experimental treatment remain possible alternatives. In addition, the subcellular localization and absolute abundance of the three RNAs were not determined, their association with Ago2-containing complexes was not examined, and no rescue experiment was performed. The lncRNA1386.1/novel-miR0032-5p/*CAT* relationship should therefore be interpreted as a candidate regulatory model compatible with ceRNA-like regulation rather than as a fully established endogenous ceRNA mechanism. Its association with *CAT* transcript abundance, CAT protein concentration, and DHE fluorescence intensity further suggests a possible link with infection-associated redox regulation.

### 4.2. The Candidate lncRNA1386.1/novel-miR0032-5p/CAT Relationship Is Associated with Redox- and Immune-Relevant Changes During Early Infection

In this study, we observed that *A. apis* infection induced a sustained increase in DHE fluorescence intensity in the larval gut from 1 to 3 dpi, indicating enhanced superoxide-associated oxidative signals during early infection (Figure 6A,C,E). Silencing lncRNA1386.1 and overexpression or inhibition of novel-miR0032-5p produced consistent changes in this response: lncRNA1386.1 silencing increased DHE fluorescence intensity (Figure 7A,C,E), novel-miR0032-5p overexpression also increased DHE fluorescence intensity (Figure 8A,C,E), whereas novel-miR0032-5p inhibition reduced it (Figure 8H,J,L). These changes were accompanied by corresponding alterations in *CAT* transcript abundance and CAT protein concentration. Taken together, the results support a negative regulatory role of novel-miR0032-5p in CAT-related antioxidant regulation and suggest that lncRNA1386.1 is associated with lower DHE fluorescence, potentially limiting infection-associated oxidative stress.

This pattern is consistent with previous studies showing that *A. apis* infection alters CAT and other antioxidant enzyme activities and is associated with oxidative-stress-related metabolic changes in honey bee larvae [6,18]. CAT removes hydrogen peroxide and thereby contributes to the balance between antimicrobial oxidant production and host tissue protection. Here, reduced *CAT* transcript abundance and CAT protein concentration were generally accompanied by stronger DHE fluorescence, while novel-miR0032-5p inhibition produced the opposite pattern. The temporal alterations in *CAT* transcript abundance, CAT protein concentration, and DHE fluorescence were not completely synchronous, which is biologically plausible because transcription, protein accumulation, oxidant production, and antioxidant clearance occur on different timescales. Other oxidant-producing enzymes, mitochondrial metabolism, and antioxidant systems are also likely to contribute to the observed oxidative response. Hence, these findings support CAT-related redox regulation within this candidate regulatory relationship, although direct CAT-dependent causality will require CAT activity measurements and functional rescue.

In addition, perturbation of lncRNA1386.1 and novel-miR0032-5p also produced coordinated changes in *Dorsal*1 and *Relish* transcript abundance. *Dorsal*1 and *Relish* transcript abundance changed in a treatment- and dpi-dependent manner following lncRNA1386.1 silencing (Figure 5A,D) or novel-miR0032-5p overexpression(Figure 5B,E) and decreased following novel-miR0032-5p inhibition (Figure 5C,F). These reciprocal responses indicate that the candidate regulatory relationship is linked not only to CAT-related antioxidant regulation but also to immune-related transcription during *A. apis* infection. *Dorsal*1 and *Relish* are central NF-κB-family regulators associated with Toll- and Imd-related antimicrobial peptide expressions in honey bees [11,12]. Their responses are therefore consistent with an adjustment of innate immune transcription under altered redox conditions.

ROS-related signals can act both as antimicrobial effectors and as modulators of immune-related transcription in insects. One possible interpretation is that reduced CAT-related antioxidant protection increases oxidative pressure in the infected gut, which in turn promotes *Dorsal*1 and *Relish*-associated transcriptional responses. Conversely, novel-miR0032-5p inhibition may preserve *CAT* expression, reduce oxidative pressure, and moderate these immune-related transcriptional responses. This interpretation links the observed molecular and physiological changes into a biologically coherent model in which ncRNA regulation contributes to the coordination of antioxidant defense and immune-related transcription. Because *Dorsal*1 and *Relish* were measured at the transcript level, the present conclusion concerns immune-related transcript changes rather than complete functional activation of the Toll or Imd pathways.

Overall, these experimental data support a model in which infection-associated changes in lncRNA1386.1 and novel-miR0032-5p influence CAT-related antioxidant regulation and thereby accompany changes in the oxidative environment and immune-related transcript abundance in the larval gut. This relationship provides a possible regulatory association between ncRNA regulation, redox homeostasis, and host responses during early *A. apis* infection.

### 4.3. Perturbation of the Host ncRNA-Redox Regulatory Relationship Is Accompanied by Changes in Selected A. apis Genes

In *A. apis*-infected larvae, *STE*11*-like* transcript abundance was altered following lncRNA1386.1 silencing and novel-miR0032-5p overexpression or inhibition at selected dpi (Figure 5J–L). These treatment- and dpi-dependent changes indicate that perturbation of the candidate host ncRNA-related regulatory relationship was accompanied by altered transcript abundance of a fungal MAPK-related signaling component. In *Saccharomyces cerevisiae*, *STE*11 participates in signaling connections among the high-osmolarity glycerol, pheromone-response, and cell-wall-integrity pathways [36]. Functional studies in *Bipolaris sorokiniana* and *Sclerotinia sclerotiorum* have further linked *STE*11 orthologs to fungal growth and development, and to oxidative-stress tolerance and pathogenicity, respectively [37,38]. The induction of *STE*11*-like* in *A. apis* is therefore consistent with transcriptional adjustment to changes in the larval-gut environment, including the increased oxidative pressure indicated by DHE fluorescence (Figure 7A,C,E and Figure 8A,C,E).

*Chit*3 presented the opposite transcriptional response. Its relative transcript abundance decreased following lncRNA1386.1 silencing or novel-miR0032-5p overexpression, whereas novel-miR0032-5p inhibition increased *Chit*3 expression (Figure 5G–I). Previous work has reported stage-dependent *AaChit*3 expression during *A. apis* infection [39], supporting its interpretation as an infection-responsive fungal transcript. The reciprocal responses of *Chit*3 and *STE*11*-like* indicate that host regulatory perturbation was accompanied by gene-specific remodeling of fungal transcription rather than a uniform increase or decrease in fungal gene expression. This pattern is suggestive of differential adjustment of MAPK-relevant signaling and chitinase-associated processes during early infection. In summary, these results support a stress-associated fungal transcriptional response to changes in the host environment.

### 4.4. Larval Survival and Chalkbrood Onset Following Perturbation of the lncRNA1386.1/novel-miR0032-5p/CAT Regulatory Relationship

Perturbation of the lncRNA1386.1/novel-miR0032-5p/*CAT* regulatory relationship produced different outcomes for larval survival and chalkbrood onset. lncRNA1386.1 silencing was associated with a significantly higher larval survival probability, whereas novel-miR0032-5p overexpression and inhibition did not significantly alter larval survival (Figure 10A–C). Colony-stratified Cox regression showed that novel-miR0032-5p inhibition was associated with a significantly higher hazard of first visible external mycelial growth than the inhibitor-control treatment, whereas no significant differences were detected following lncRNA1386.1 silencing or novel-miR0032-5p overexpression (Figure 10D–F). Thus, novel-miR0032-5p inhibition affected visible external mycelial growth without producing a detectable change in larval survival.

Survival and mummification represent related but biologically distinct disease outcomes. Survival integrates mortality arising from infection, developmental failure, treatment-related stress, and other physiological disturbances, whereas mummification represents a more disease-specific endpoint of chalkbrood infection [4]. The absence of a survival difference following novel-miR0032-5p inhibition therefore does not exclude changes in the timing or phenotypic manifestation of infection. Instead, the higher hazard of first visible external mycelial growth following novel-miR0032-5p inhibition suggests that this treatment altered the timing or probability of visible chalkbrood manifestation among infected larvae.

This disease-associated response occurred together with changes in *CAT* transcript abundance and CAT protein concentration, DHE fluorescence intensity, *Dorsal*1 and *Relish* transcript abundance, and selected fungal transcripts. The combined pattern supports a functional link between novel-miR0032-5p-mediated regulation and the physiological environment in which chalkbrood-associated mummification develops. One possible explanation is that changes in antioxidant regulation and host-fungus interactions influenced the emergence of visible external mycelia following novel-miR0032-5p inhibition, whereas larval mortality in this experiment remained governed by multiple overlapping processes. Because fungal biomass, internal hyphal development, tissue pathology, apoptosis, and the immediate causes of larval death were not measured, the present findings establish an association with visible external mycelial growth rather than the mechanism responsible for this phenotype.

### 4.5. Limitations and Future Directions

This study combined sequence-responsive reporter assays, in vivo RNA perturbation, *CAT* transcript and CAT protein measurements, DHE fluorescence imaging, host and fungal transcript analyses, and longitudinal infection-related outcomes. This integrated design provided multi-level evidence for an infection-responsive lncRNA1386.1/novel-miR0032-5p/*CAT* regulatory relationship compatible with a ceRNA-like model. However, endogenous ceRNA competition was not directly examined through absolute RNA-abundance measurements, subcellular co-localization, Ago2/RISC association, or rescue and epistasis experiments. These approaches will be important for determining whether lncRNA1386.1 regulates *CAT* through competition for novel-miR0032-5p in the larval gut.

Visible chalkbrood manifestation was scored using the predefined criterion of external fungal mycelial growth on dead larvae, whereas absolute fungal burden, hyphal development, histopathological changes, apoptosis, and host metabolic status were not quantified. Independent infection experiments involving fungal-load measurements and pathological staging would strengthen the connection between the molecular responses identified here and chalkbrood-associated phenotypes.

## 5. Conclusions

In conclusion, this study identifies an infection-responsive regulatory relationship among lncRNA1386.1, novel-miR0032-5p, and *CAT* in *Apis cerana* worker larvae during *A. apis* infection. This relationship was associated with changes in *CAT* transcript abundance, CAT protein concentration, DHE-derived fluorescence, host immune-related transcripts, selected fungal transcripts, larval survival, and visible external mycelial growth. These data support a negative regulatory role of novel-miR0032-5p in *CAT* expression and indicate that lncRNA1386.1 participates in this regulatory relationship. lncRNA1386.1 silencing was associated with increased larval survival, whereas novel-miR0032-5p overexpression and inhibition did not significantly alter survival. In addition, novel-miR0032-5p inhibition was associated with a higher hazard of first visible external mycelial growth.

## Data Availability

The raw data supporting the conclusions of this article are provided in the Supplementary Materials. Further inquiries can be directed at the corresponding authors.

## Supplementary Materials

The following supporting information can be downloaded at: https://www.mdpi.com/article/10.3390/antiox15080959/s1.

## References

[1] Koetz A.H. (2013). Ecology, behaviour and control of *Apis cerana* with a focus on relevance to the Australian incursion. Insects.

[2] Chen D., Guo R., Xiong C., Zheng Y., Hou C., Fu Z. (2018). Morphological and molecular identification of chalkbrood disease pathogen *Ascosphaera apis* in *Apis cerana cerana*. J. Apic. Res..

[3] Castagnino G.L.B., Mateos A., Meana A., Montejo L., Zamorano Iturralde L.V., Cutuli De Simón M.T. (2020). Etiology, symptoms and prevention of chalkbrood disease: A literature review. Rev. Bras. Saúde Produção Anim..

[4] von Knoblauch T., Jensen A.B., Mülling C.K.W., Aupperle-Lellbach H., Genersch E. (2024). Chalkbrood disease caused by *Ascosphaera apis* in honey bees (*Apis mellifera*)—Morphological and histological changes in infected larvae. Vet. Sci..

[5] Wu Y., Guo Y., Fan X., Zhao H., Zhang Y., Guo S., Jing X., Liu Z., Feng P., Liu X. (2023). Ame-mir-34 modulates the larval body weight and immune response of *Apis mellifera* workers to *Ascosphaera apis* invasion. Int. J. Mol. Sci..

[6] Zhang K., Fu Z., Fan X., Wang Z., Wang S., Guo S., Gao X., Zhao H., Jing X., Zou P. (2023). Effect of *Ascosphaera apis* infestation on the activities of four antioxidant enzymes in Asian honey bee larval guts. Antioxidants.

[7] Evans J.D., Aronstein K., Chen Y.P., Hetru C., Imler J.-L., Jiang H., Kanost M., Thompson G.J., Zou Z., Hultmark D. (2006). Immune pathways and defence mechanisms in honey bees *Apis mellifera*. Insect Mol. Biol..

[8] Lemaitre B., Nicolas E., Michaut L., Reichhart J.-M., Hoffmann J.A. (1996). The dorsoventral regulatory gene cassette spätzle/Toll/cactus controls the potent antifungal response in *Drosophila* adults. Cell.

[9] Weber A.N.R., Tauszig-Delamasure S., Hoffmann J.A., Lelièvre E., Gascan H., Ray K.P., Morse M.A., Imler J.-L., Gay N.J. (2003). Binding of the *Drosophila* cytokine Spätzle to Toll is direct and establishes signaling. Nat. Immunol..

[10] Valanne S., Wang J.H., Rämet M. (2011). The *Drosophila* Toll signaling pathway. J. Immunol..

[11] Lourenço A.P., Florecki M.M., Simões Z.L.P., Evans J.D. (2018). Silencing of *Apis mellifera dorsal* genes reveals their role in expression of the antimicrobial peptide defensin-1. Insect Mol. Biol..

[12] Schlüns H., Crozier R.H. (2007). *Relish* regulates expression of antimicrobial peptide genes in the honeybee, *Apis mellifera*, shown by RNA interference. Insect Mol. Biol..

[13] Ha E.-M., Lee K.-A., Seo Y.-Y., Kim S.-H., Lim J.-H., Oh B.-H., Kim J., Lee W.-J. (2009). Coordination of multiple dual oxidase-regulatory pathways in responses to commensal and infectious microbes in the *Drosophila* gut. Nat. Immunol..

[14] Buchon N., Broderick N.A., Chakrabarti S., Lemaitre B. (2009). Invasive and indigenous microbiota impact intestinal stem cell activity through multiple pathways in *Drosophila*. Genes Dev..

[15] Ha E.-M., Oh C.-T., Bae Y.-S., Lee W.-J. (2005). A direct role for dual oxidase in *Drosophila* gut immunity. Science.

[16] Schieber M., Chandel N.S. (2014). ROS function in redox signaling and oxidative stress. Curr. Biol..

[17] Chelikani P., Fita I., Loewen P.C. (2004). Diversity of structures and properties among catalases. Cell. Mol. Life Sci..

[18] Li Z., Hou M., Qiu Y., Zhao B., Nie H., Su S. (2020). Changes in antioxidant enzymes activity and metabolomic profiles in the guts of honey bee (*Apis mellifera*) larvae infected with *Ascosphaera apis*. Insects.

[19] Wang Z. (2018). Diverse roles of regulatory non-coding RNAs. J. Mol. Cell Biol..

[20] Rojas-Pirela M., Andrade-Alviárez D., Quiñones W., Rojas M.V., Castillo C., Liempi A., Medina L., Guerrero-Muñoz J., Fernández-Moya A., Ortega Y.A. (2023). microRNAs: Critical players during helminth infections. Microorganisms.

[21] Ma L., Liu L., Zhao Y., Yang L., Chen C., Li Z., Lu Z. (2020). JNK pathway plays a key role in the immune system of the pea aphid and is regulated by microRNA-184. PLoS Pathog..

[22] Zhou H., Huang Y., Jia C., Pang Y., Liu L., Xu Y., Jin P., Qian J., Ma F. (2024). NF-κB factors cooperate with Su(Hw)/E4F1 to balance *Drosophila*/human immune responses via modulating dynamic expression of miR-210. Nucleic Acids Res..

[23] Song Y., Qiu J., Kang J., Chen Y., Cao R., Wang W., Dai M., Chen D., Fu Z., Guo R. (2025). Transcriptomic characterization of miRNAs in *Apis cerana* larvae responding to infection. Genes.

[24] Zafar J., Huang J., Xu X., Jin F. (2022). Analysis of long non-coding RNA-mediated regulatory networks of *Plutella xylostella* in response to *Metarhizium anisopliae* Infection. Insects.

[25] Guo R., Wang S., Guo S., Fan X., Zang H., Gao X., Jing X., Liu Z., Na Z., Zou P. (2023). Regulatory roles of long non-coding RNAs relevant to antioxidant enzymes and immune responses of *Apis cerana* larvae following *Ascosphaera apis* invasion. Int. J. Mol. Sci..

[26] Salmena L., Poliseno L., Tay Y., Kats L., Pandolfi P.P. (2011). A ceRNA hypothesis: The Rosetta stone of a hidden RNA language?. Cell.

[27] Denzler R., Agarwal V., Stefano J., Bartel D.P., Stoffel M. (2014). Assessing the ceRNA hypothesis with quantitative measurements of miRNA and target abundance. Mol. Cell.

[28] Bosson A.D., Zamudio J.R., Sharp P.A. (2014). Endogenous miRNA and target concentrations determine susceptibility to potential ceRNA competition. Mol. Cell.

[29] Calloni R., Bonatto D. (2019). Characteristics of the competition among RNAs for the binding of shared miRNAs. Eur. J. Cell Biol..

[30] Guo R., Chen D., Diao Q., Xiong C., Zheng Y., Hou C. (2019). Transcriptomic investigation of immune responses of the *Apis cerana cerana* larval gut infected by *Ascosphaera apis*. J. Invertebr. Pathol..

[31] Guo R., Zhang K., Zang H., Guo S., Liu X., Jing X., Song Y., Li K., Wu Y., Jiang H. (2024). Dynamics and regulatory role of circRNAs in Asian honey bee larvae following fungal infection. Appl. Microbiol. Biotechnol..

[32] Schindelin J., Rueden C.T., Hiner M.C., Eliceiri K.W. (2015). The ImageJ ecosystem: An open platform for biomedical image analysis. Mol. Reprod. Dev..

[33] Livak K.J., Schmittgen T.D. (2001). Analysis of relative gene expression data using real-time quantitative PCR and the 2^−ΔΔCT^ method. Methods.

[34] Cesana M., Cacchiarelli D., Legnini I., Santini T., Sthandier O., Chinappi M., Tramontano A., Bozzoni I. (2011). A long noncoding RNA controls muscle differentiation by functioning as a competing endogenous RNA. Cell.

[35] Tay Y., Rinn J., Pandolfi P.P. (2014). The multilayered complexity of ceRNA crosstalk and competition. Nature.

[36] Leng G., Song K. (2016). Direct interaction of Ste11 and Mkk1/2 through Nst1 integrates high-osmolarity glycerol and pheromone pathways to the cell wall integrity MAPK pathway. FEBS Lett..

[37] Leng Y., Zhong S. (2015). The role of mitogen-activated protein (MAP) kinase signaling components in the fungal development, stress response and virulence of the fungal cereal pathogen *Bipolaris sorokiniana*. PLoS ONE.

[38] Tian L., Li J., Xu Y., Qiu Y., Zhang Y., Li X. (2024). A MAP kinase cascade broadly regulates the lifestyle of *Sclerotinia sclerotiorum* and can be targeted by HIGS for disease control. Plant J..

[39] Wang M., Wu T., Fan X., Hu Y., Wang W., Zheng Y., Qiu J., Zhang R., Yan T., Chen D. (2025). Bioinformatics and expression pattern analysis of the *Chitinase* 3 gene in *Ascosphaera apis*. J. Fujian Agric. For. Univ. (Nat. Sci. Ed.).

