# Supplementary material for: lncRNA1386.1/novel-miR0032-5p Axis Targets CAT Gene to Modulate Redox and Immune Reponses of Apis cerana Larvae to Ascosphaera apis Infection
Source: Antioxidants (Basel). 2026 Jul 31;15(8):959. doi: 10.3390/antiox15080959 (PMC13509846; doi:10.3390/antiox15080959)
Supplement: Supplementary file 1 [file antioxidants-15-00959-s001.zip › Table S1.pdf]

Table S1. qRT-PCR validation information, primer sequences used in this study

**Part A. Sequences of primers, mimics, inhibitors, and siRNAs used in this study**

| Name | Sequence (5'-3') |
|---|---|
| novel-miR0032-5p-loop | GTCGTATCCAGTGCAGGGTCCGAGGTATTCGCACTGGATACG ACAGACGA |
| novel-miR0032-5p-F | GCGCGCGTTAATTAAATACATCT |
| novel-miR0032-5p-R | AGTGCAGGGTCCGAGGTATT |
| lncRNA1386.1-F | GGAGCGTTGAGACACTATGG |
| lncRNA1386.1-R | GCCTATCATCTATCACCGTTCT |
| CAT-F | ATAGAGGTATACCCGATGGT |
| CAT-R | GGCGGCTGAATTAAGTGCTA |
| Relish-F | TACTGATGGTCCTTCAACTGTG |
| Relish-R | GTGCTGCATGTAATGGAGAATC |
| Dorsal1-F | AATGTGCCACTACAACCA |
| Dorsal1-R | TGCGTTACTATGACTGAGTT |
| Chit3-F | CTGAGCCCACCACCACAT |
| Chit3-R | CCCGTTGTTCTTGTAGATTGTC |
| STE11-like-F | TCCTACTGCTACACCTGAC |
| STE11-like-R | TAGAATACTGAGTGGTTGAGAG |

| | |
|---|---|
| U6-F | GTTAGGCTTTGACGATTTCG |
| U6-R | GGCATTTCTCCACCAGGTA |
| actin-F | TTATATGCCAACACTGTCCTTT |
| actin-R | AGAATTGATCCACCAATCCA |
| 5.8s rRNA-F | GGCACTTTGTTAGGCTTTG |
| 5.8s rRNA-R | GTTTGGTGACTCTTCTTCCTTC |
| CAT-wt-F | CTTATTATACGATTATTGATGTTCAATTAATTATGTAAAC |
| CAT-wt-R | TCGAGTTTACATAATTAATTGAACATCAATAATCGTATAATAA GAGCT |
| CAT-mut-F | CTTATTACGATGCCGCCTGCTCCAGGCCGGCCGTGTAAAC |
| CAT-mut-R | TCGAGTTTACACGGCCGGCCTGGAGCAGGCGGCATCGTAATA AGAGCT |
| lncRNA1386.1-wt-F | CCGCTATTCGAGGATAATTTAATTATCCTTCAC |
| lncRNA1386.1-wt-R | TCGAGTGAAGGATAATTAAATTATCCTCGAATAGCGGAGCT |
| lncRNA1386.1-mut-F | CCGCTATCATGTTGCGGCCCGGCCGCCCTTCAC |
| lncRNA1386.1-mut-R | TCGAGTGAAGGGCGGCCGGGCCGCAACATGATAGCGGAGCT |
| M-miR0032-5p-sense | UUAAUUAAAUACAUCUUCGUCU |
| M-miR0032-5p-antisense | ACGAAGAUGUAUUUAAUUAAUU |
| M-NC-sense | UUCUCCGAACGUGUCACGUTT |

| | |
|---|---|
| M-NC-antisense | ACGUGACACGUUCGGAGAATT |
| I-miR0032-5p | AGACGAAGAUGUAUUUAAUUAA |
| I-NC | CAGUACUUUUGUGUAGUACAA |
| si-lncRNA1386.1-sense | GGACACAUCUACUUGCGUU |
| si-lncRNA1386.1-antisense | AACGCAAGUAGAUGUGUCC |
| si-scramble-sense | UUCUCCGAACGUGUCACGUTT |
| si-scramble-antisense | ACGUGACACGUUCGGAGAATT |

**Part B. qPCR amplification performance**

| Primer | $R^2$ | Efficiency (%) | Melt curve |
|---|---|---|---|
| lncRNA1386.1 | 0.996 | 97.4 | Single peak |
| CAT | 0.998 | 96.7 | Single peak |
| novel-miR0032-5p | 0.998 | 98.6 | Single peak |
| Relish | 0.997 | 99.1 | Single peak |
| Dorsal1 | 0.997 | 97.9 | Single peak |
| Chit3 | 0.998 | 98.6 | Single peak |
| STE11-like | 0.997 | 98.6 | Single peak |
| U6 | 0.997 | 96.6 | Single peak |
| actin | 0.999 | 100.3 | Single peak |
| 5.8s rRNA | 0.997 | 101.1 | Single peak |
